# Preclinical Development and Clinical-Scale Manufacturing of HIV Gag-Specific, LentivirusModified CD4 T Cells for HIV Functional Cure

**DOI:** 10.1016/j.omtm.2020.04.024

**Published:** 2020-05-03

**Authors:** Haishan Li, Tyler Lahusen, Lingzhi Xiao, Nidal Muvarak, Jana Blazkova, Tae-Wook Chun, C. David Pauza

**Affiliations:** 1 American Gene Technologies International, Rockville, MD, USA; 2 Laboratory of Immunoregulation, National Institute of Allergy and Infectious Diseases (NIAID), Bethesda, MD, USA

**Keywords:** HIV, AIDS, functional cure, immunotherapy, lentivirus vector, autologous, CD4 T cell, gene therapy, NCT03215004

## Abstract

Activation, infection, and eventual depletion of human immunodeficiency virus (HIV)-specific cluster of differentiation 4 (CD4) T cells are the crucial pathogenetic events in acquired immunodeficiency syndrome (AIDS). We developed a cell and gene therapy to reconstitute HIV-specific CD4 T cells and prevent their destruction by HIV. Antigen-specific CD4 T cells will provide helper functions to support antiviral cytotoxic T lymphocyte (CTL) function and the production of virus-specific antibodies. However, *ex vivo* expansion of HIV-specific CD4 T cells is poor and previous gene therapies focused on bulk CD4 T cells without enriching for an antigen-specific subset. We developed a method for manufacturing autologous CD4^+^ T cell products highly enriched with Gag-specific T cells. Rare Gag-specific CD4 T cells in peripheral blood mononuclear cells (PBMCs) were increased nearly 1,000-fold by stimulating PBMC with Gag peptides, followed by depleting nontarget cells and transducing with lentivirus vector AGT103 to protect against HIV-mediated depletion and inhibit HIV release from latently infected cells. The average percentage of HIV-specific CD4 cells in the final products was 15.13%, and the average yield was 7 × 10^8^ cells. The protocol for clinical-scale manufacturing of HIV-specific and HIV-resistant CD4 T cells is an important step toward effective immunotherapy for HIV disease.

## Graphical Abstract

**Figure:**
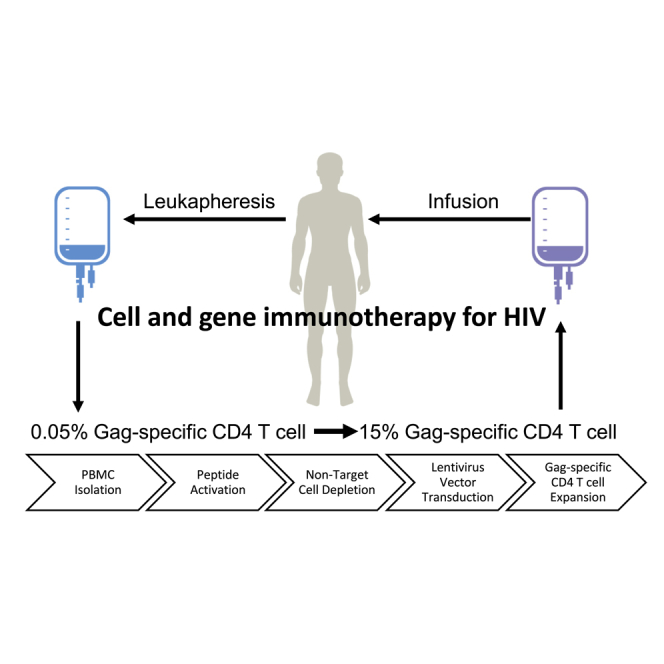

## Teaser

Cell and gene therapy can reconstitute HIV-specific CD4 T cells and correct the key defect in HIV disease. A new process enriches for Gag-specific CD4^+^ T cells from HIV-infected individuals to an average of 15% with yields nearing 10^9^-specific cells per product and may improve antiviral immunity. Keywords: HIV; AIDS; functional cure; immunotherapy; lentivirus vector; autologous; CD4 T cell; gene therapy; NCT03215004

## Introduction

HIV infection is a chronic disease characterized by ongoing viral replication with gradual exhaustion and destruction of cluster of differentiation 4 (CD4) T lymphocytes. Antiretroviral therapy (ART) can suppress viremia and prolong life but is not without side effects and does not cure the disease. The recent developments of autologous T cell therapies for cancer, chronic viral infection, and viral reactivation post-transplant^1^, ^2^, ^3^, ^4^, ^5^, ^6^, ^7^ suggest that a similar strategy might be used to control HIV and reduce the dependence on ART.^8^ A consistent feature of successful T cell therapies is the enrichment of antigen-specific cells, either through amplification after antigen stimulation or by introducing new antigen receptors to redirect the T cell response. Our strategy is to reconstitute the CD4 T cell population by infusing HIV-specific CD4 T cells that will be durable within the HIV-infected individual due to the introduction of a lentivirus vector that modulates C-C chemokine receptor type 5 (CCR5) levels and decreases the abundance of HIV mRNA and genomic RNA. A substantial obstacle to creating such an autologous cell product is poor growth in culture of HIV-specific CD4 T cells. We devised an efficient process for manufacturing autologous CD4 T cells from HIV-infected individuals that is both specific for HIV and resistant to viral-mediated depletion.

We elected to enrich HIV-specific CD4^+^ T cells because they are targeted for depletion by HIV, the levels of HIV-specific CD4 T cell function correlate with clinical status, and degeneracy of major histocompatibility complex (MHC) class II-restricted T cell receptor (TCR) recognition meant we did not need to define autologous virus sequences to stimulate CD4 T cells from individual, HIV^+^ persons. We know that virus-specific CD8 T cells have been successful for treating some virus-associated cancers and viral reactivation in the post-transplant scenario.^5^^,^^9^, ^10^, ^11^, ^12^ Treatment with HIV-specific CD8 T cell therapy was safe but provided only a transient decrease in viral burden consistent with rapid elimination of the transferred cells. In other examples of infection, there are important roles for antigen-specific CD4 T cells. Adenovirus infection poses a significant risk for morbidity and mortality in children undergoing hematopoietic stem cell transplantation.^13^^,^^14^ These children were treated successfully with donor-derived peripheral blood mononuclear cells (PBMCs) that had been stimulated *ex vivo* with adenovirus hexon protein.^15^ The elimination of adenovirus DNA depended on a strong, antigen-specific CD4 T cell response that was needed to amplify the population of effector CD8 T cells.^16^

The paucity of HIV-specific CD4 T cells may be one reason why CD8 T cell therapy has been unsuccessful in HIV disease. CD4 T cells isolated during acute HIV infection can support *ex vivo* proliferation of HIV-specific CD8 T cells from chronically infected individuals, and loss of HIV-specific CD8 T cell proliferation after acute HIV infection was restored *in vivo* by infusing vaccine-induced, HIV-specific CD4^+^ T cells.^17^ In HIV elite controllers, *in vitro* peptide-stimulated proliferation of virus-specific CD8 T cells was abrogated when CD4 T cells were depleted, showing that CD4 T cells are necessary to sustain the anti-HIV CD8 T cell responses.^18^ We also know that CD4 T cells are crucial for orchestrating a number of immune responses to viral infection. Thus, antigen-specific CD4 T cells provide help to promote expansion and acquisition of effector function for both CD8 T cells and B cells; they may also manifest MHC class II-restricted cell-mediated cytotoxicity,^19^ which is important for clearing persistent viral infections.^4^

The primary pathogenic mechanism of HIV is dysregulation of host immunity characterized by generalized, nonspecific immune activation and depletion of CD4 T cells. Reduced CD4 T cells and especially the near-complete destruction of CD4 T cells specific for HIV antigens disable the antiviral immune response and allow HIV to persist. As HIV sequences drift to evade host responses, the immune system depleted of CD4 T cells no longer has the capacity to generate *de novo* CD8 T cell responses against changing epitopes, and the virus grows unchecked. The restoration of strong CD4 T cell immunity against HIV is needed to support the continuing evolution of T and B cell responses needed to reconstitute normal immune control of this viral disease.

The development of CD4 T cell therapy for HIV infection requires approaches different from those used for other viruses and cancers. As a target of HIV, CD4 T cells must be modified to resist HIV infection before being used for therapy. Several efforts have focused on disrupting or deleting the coreceptors for HIV, CCR5, and C-X-C chemokine receptor type 4 (CXCR4) through gene-editing strategies intended to prevent viral entry into CD4 T cells.^20^, ^21^, ^22^, ^23^ Clinical studies evaluated the safety and efficacy of infusing CD4 T cells with zinc finger nuclease (ZFN)-targeted disruption of the CCR5 gene (see ClinicalTrials.gov: NCT00842634, NCT01252641, and NCT01044654). Published results from the University of Pennsylvania^22^ and information released by Sangamo Biotherapeutics showed safety and modest HIV suppression after infusing participants with CCR5-modified, autologous CD4 T cells, but successful control of viremia was only achieved in a trial participant who is heterozygous for the null allele CCR5Δ32.^22^

Vigorous HIV-specific CD4 T cell responses are associated with efficient control of viremia.^18^^,^^24^ HIV controllers exhibit more robust HIV-specific CD4 T cell responses compared to individuals with progressive, untreated infection.^25^ Among elite controllers, HIV-specific cytotoxic CD4 T cell levels correlate with viral suppression.^26^, ^27^, ^28^ Due to CD4 T cell dysregulation in most individuals with HIV infection and the failure to restore antigen-specific memory CD4 T cells even after years of virus-suppressive antiretroviral therapy, it is particularly important to provide a therapeutic reconstitution of antigen-specific CD4 T cells as a means for re-establishing immunity against HIV. To date, there have been few published studies on HIV-specific CD4 T cell therapy. This might be due to technical difficulties in obtaining sufficient HIV-specific and HIV-resistant CD4 T cells to impart a therapeutic effect. In this study, we developed and optimized a protocol for efficient clinical-scale manufacturing of a cell product enriched for polyclonal, HIV-specific CD4 T cells that resists HIV destruction due to protective effects of a therapeutic lentivirus vector. This cell product, AGT103-T, is intended to be an autologous cell therapy, delivering CD4 T cells that are specific for the HIV Gag protein and capable of surviving and promoting antiviral immunity even in the presence of infectious virus.

## Results

### Construction and Evaluation of Lentivirus AGT103 for Blocking HIV Infection and Replication

**Figure 1:**
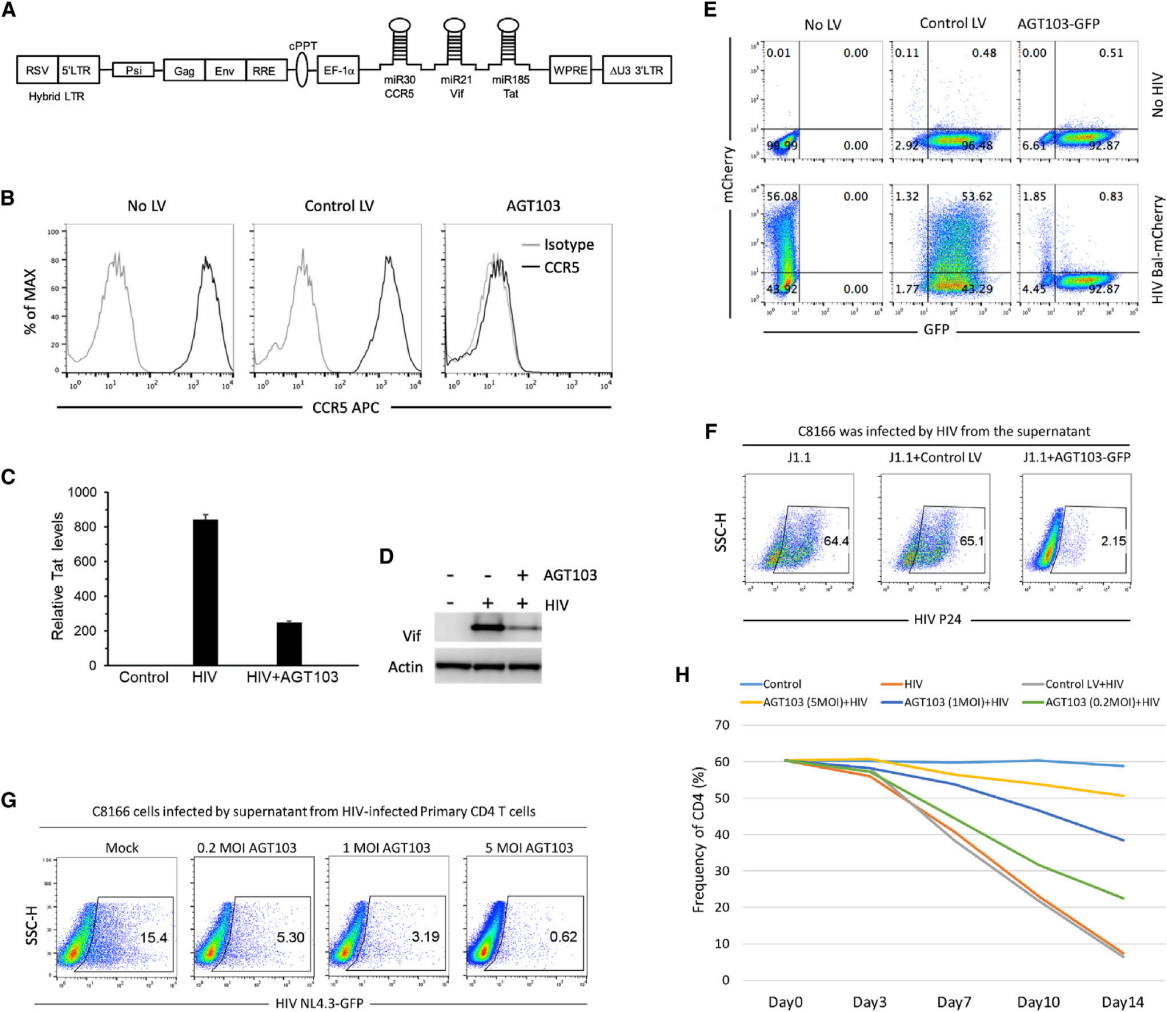
Construction and Evaluation of Lentivirus AGT103 (A) Schematic diagram of the lentiviral vector AGT103. (B) JC53 cells were transduced with AGT103 or a control lentivirus vector. CCR5 expression was examined 6 days later by flow cytometry (see also Figures S1 and S2). The horizontal axis depicts Mean Fluorescence Intensity (MFI) and the vertical axis depicts the percentage of maximum cell count. (C and D) 293 T cells were transduced without or with AGT103 for 48 h before transfecting the HIV proviral vector pNL4.3-GFP. After 24 h, (C) RNA was extracted and Tat expression levels were determined by real-time PCR (results are mean values of 3 replicates, + Standard Deviation), and (D) cells were lysed and protein lysates were analyzed by immunoblot. (E) JC53 cells were transduced with AGT103-GFP or a control lentivirus vector carrying GFP and infected with a R5 tropic BaL HIV virus carrying mCherry. LV transduction and HIV infection were detected by flow cytometry assay. The horizontal axis depicts fluorescence intensity for the GFP marker and the vertical axis depicts fluorescence intensity for the mCherry marker. Values indictate the percentage of total cells in each sector. (F) J1.1 cells were transduced with AGT103-GFP or a control lentivirus vector carrying GFP and detected by flow cytometry. Transduced J1.1 cells were treated with TNF-α (50 ng/mL) to induce HIV production. Supernatants were collected after 3 days to infect HIV-permissive C8166 cells. HIV infection was detected by intracellular p24 staining and flow cytometry assay. The horizontal axis depicts fluorescence intensity for staining of Gag (p24) protein and the vertical axis depicts side scatter height. (G) CD4 T cells separated from PBMCs were stimulated with CD3/CD28 beads + IL-2 for 1 day and transduced without or with AGT103 at various concentrations. After 2 days, beads were removed, and CD4 T cells were infected with 0.1 MOI of HIV NL4.3-GFP. 24 h later, cells were washed 3 times with PBS and cultured with IL-2 (30 U/mL) for 7 days. At the end of the culture, supernatant was collected to infect the HIV-permissive cell line C8166 for 2 days. HIV-infected C8166 cells (GFP positive) were detected by flow cytometry. The horizontal axes depict intensity of green fluorescence and the vertical axes depict side scatter height. Values in the enclosed areas indicate the precentages of total cells that are positive for GFP expression. (H) PBMCs were stimulated with CD3/CD28 beads + IL-2 for 1 day and transduced without or with AGT103 at various concentrations. After 2 days, beads were removed, and cells were infected with 0.1 MOI of HIV NL4.3. 24 h later, cells were washed 3 times with PBS and cultured with IL-2 (30 U/mL). Cells were collected every 3 days, and the frequency of CD4 cells was analyzed by flow cytometry. Data are representative of three independent experiments.

We developed a recombinant lentivirus vector (designated AGT103) encoding inhibitory RNA targeting the HIV coreceptor CCR5 and HIV sequences within the Vif/Tat coding regions. A schematic of the gene-transfer vector is shown (Figure 1A). The lentivirus AGT103 expresses three inhibitory microRNAs (miRNAs) within a single transcript driven by the RNA polymerase II promoter elongation factor-1 alpha (EF-1 alpha; J04617.1). Transcription generates RNA containing three miRNA hairpin structures with targeting sequences specific for the following: (1) *Homo sapiens* *CCR5* gene (C-C chemokine receptor type 5) (GenBank: GQ917109.1), (2) HIV *TAT* gene (transactivator of transcription) (GenBank: AAK08486.1), and (3) HIV *VIF* gene (viral infectivity factor) (GenBank: AAK08482.1). A CCR5 targeting sequence is embedded within the naturally occurring human miR30, a HIV *TAT* targeting sequence is embedded within miR185, and a HIV *VIF* targeting sequence is embedded within miR21. The individual, native miRNA backbones; selected guide sequences; and order of miRNA were optimized for vector potency among many alternative structures (not shown). Modifications of the naturally occurring miRNA destroy their normal function, and the resulting synthetic versions are highly specific for the target genes listed. The RNA transcript is processed by cellular factors to produce active miRNA.

To demonstrate the effects of AGT103 on CCR5 expression, we used the CCR5-positive cell line JC53 as a model. JC53 is a modified HeLa cell line that stably expresses high levels of both CD4 and CCR5.^29^ JC53 cells were transduced with AGT103 or a control lentivirus, and cell-surface CCR5 expression was examined by flow cytometry. At a multiplicity of infection (MOI) equal to 5, AGT103 reduced CCR5 levels on JC53 cells by more than 98% (Figures 1B and S1).

We next tested the specificity of AGT103. The CCR2 and CCR5 coding regions are approximately 75% homologous, but CCR5-specific miRNA does not target sequences shared between CCR5 and CCR2. To exclude potential adverse effects of AGT103 on CCR2, we tested the effect of AGT103 transduction in Vγ9Vδ2 T cells, which express both CCR2 and CCR5. AGT103 significantly reduced CCR5 but did not affect CCR2 expression in Vγ9Vδ2 T cells (Figure S2A). The CEM.NKR.CCR5 cell line was then used to test for effects of AGT103 on common cell-surface proteins, including CD4, CD5, CCR7, CXCR4, CXCR5, and α4 integrin. As shown (Figure S2B), AGT103 specifically decreased CCR5 expression without affecting expression levels for any of the listed cell-surface molecules.

To test whether AGT103 inhibits HIV Tat and Vif expression, 293T cells were transduced with AGT103 or control lentivirus vector for 48 h before transfecting with the HIV proviral vector pNL4.3. After 24 h, RNA was extracted, and Tat RNA levels were measured by real-time RT-PCR (Figure 1C). To measure Vif expression, we prepared protein lysates from transduced cells and analyzed Vif protein levels by immunoblotting (Figure 1D). The AGT103 vector significantly inhibited expression of HIV Tat RNA (Figure 1C) and Vif protein (Figure 1D).

To determine whether AGT103-modified cells resist CCR5-tropic HIV infection, JC53 cells were transduced with AGT103-GFP. We incorporated a GFP construct expressed under a separate promoter into AGT103 or into a control lentivirus vector to facilitate identification of transduced cells. 6 days after transduction, cell cultures were infected with R5-tropic BaL HIV pseudovirus carrying mCherry as a marker. 2 days later, lentivirus transduction (Figure 1E; GFP-positive, horizontal axes) and HIV infection (Figure 1E; mCherry-positive, vertical axes) were detected by flow cytometry. Among JC53 cells that were not transduced, 56% became infected by HIV (Figure 1E; no lentiviral [LV]). Transducing JC53 cells with a control lentivirus vector then challenging with HIV resulted in 54% of cells becoming infected (Figure 1E; control LV), indicating that control lentivirus vector did not inhibit HIV infection. Transducing JC53 cells with AGT103 greatly reduced the frequency of double-positive cells to 0.83%, and the vast majority of GFP-positive cells (93%) were protected from HIV infection (Figure 1E; AGT103-GFP). Our AGT103 vector blocked more than 90% of CCR5-tropic HIV infection compared with a control lentivirus (Figure 1E).

We then tested whether miRNA against Vif and Tat inhibited infectious HIV production in cells that were already infected by HIV. We used the J1.1 cell line as a model.^30^ J1.1 is a HIV latently infected cell line cloned by limiting dilution from HIV-infected Jurkat cells. HIV replication in J1.1 can be induced by treating with tumor necrosis factor alpha (TNF-α). In this study, J1.1 cells were transduced with AGT103-GFP or a control lentivirus vector-GFP at MOI 5. 1 day after transduction, culture medium was removed and replaced with fresh medium; 2 days later, control or transduced cells were treated with recombinant human TNF-α (50 ng/mL) and cultured for an additional 3 days. At the end of the culture period, supernatants containing HIV particles were collected and used to infect the permissive cell line C8166 (Figure 1F). 2 days after transferring culture fluids, C8166 cells were collected and analyzed by flow cytometry to detect intracellular HIV p24.

Supernatant from control J1.1 caused 64.4% of C8166 cells to be infected by HIV (Figure 1F; J1.1). Control LV did not inhibit HIV production in J1.1 cells, and 65.1% of C8166 cells became infected (Figure 1F; J1.1 + control LV). When the same test was done with supernatants from J1.1 that had been transduced with the AGT103 vector, only 2.15% of C8166 cells became infected (Figure 1F; J1.1 + AGT103-GFP). AGT103-GFP transduction into J1.1 cells substantially reduced the amount of infectious HIV released after TNF-α induction.

We also tested whether AGT103 inhibits HIV replication in primary human CD4 T cells. CD4 T cells were purified from PBMC using negative selection and stimulated for 1 day with CD3/CD28 Dynabeads (nanobeads coated with antibodies against CD3 and CD28) plus interleukin 2 (IL-2; 30 U/mL) and then transduced with AGT103 or a control lentivirus at various MOI. 2 days after transduction, cells were infected with 0.1 MOI of HIV strain NL4.3 that itself expresses GFP and importantly, uses the CXCR4 coreceptor. This assay measures the direct antiviral effects of AGT103 irrespective of coreceptor modulation, since HIV NL4.3 does not require CCR5 for cellular attachment and penetration.

At the end of the culture, supernatants containing HIV particles were collected and added to C8166 cells. 2 days later, C8166 cells were collected and analyzed by flow cytometry to detect GFP that was expressed by the NL4.3-GFP virus. AGT103 protected primary CD4 T cells against HIV replication in a dose-dependent manner (Figure 1G). At the lowest AGT103 dose, culture fluids contained enough HIV to infect approximately 5% of C8166 cells, and that value was well below the 15% infection of C8166 cells that were observed with a control lentivirus vector (Figure 1G; mock). At the highest AGT103 dose of 5 MOI, culture fluids contained little HIV and only infected 0.6% of C8166 cells (Figure 1G; 5 MOI AGT103).

We next tested the capacity for AGT103 to protect primary human CD4 T cells against challenge with infectious HIV. Fresh PBMC were stimulated for 1 day with CD3/CD28 Dynabeads plus IL-2 (30 U/mL) and then transduced with AGT103 (or mock) at various concentrations. 2 days later, cells were challenged by infecting with 0.1 MOI of HIV NL4.3. Cell samples were collected every 3 days, and the frequency of CD4 cells was determined by flow cytometry. Uninfected CD4 cells comprised approximately 60% of total cells in culture and remained at these values for 14 days (Figure 1H; control). Untreated or mock-transduced CD4 T cells were depleted rapidly after HIV challenge, falling from a starting value of around 60% of cells in culture to <10% of total cells by day 14 (Figure 1H; HIV and control LV + HIV). Transduction with AGT103 showed strong, dose-dependent protection of CD4 T cells against infection with HIV NL4.3. By day 14, the 0.2 MOI dose of AGT103 preserved CD4 T cells at levels >20% of cells in culture, and the highest AGT103 dose of MOI 5 preserved CD4 T cells at >50% of cells in culture (Figure 1H; AGT103 + HIV). The results established that AGT103 was potent for protecting primary CD4 T cells during 14 days in culture.

### Developing an Optimized Protocol for Expanding HIV Gag-Specific CD4 T Cells

**Figure 2:**
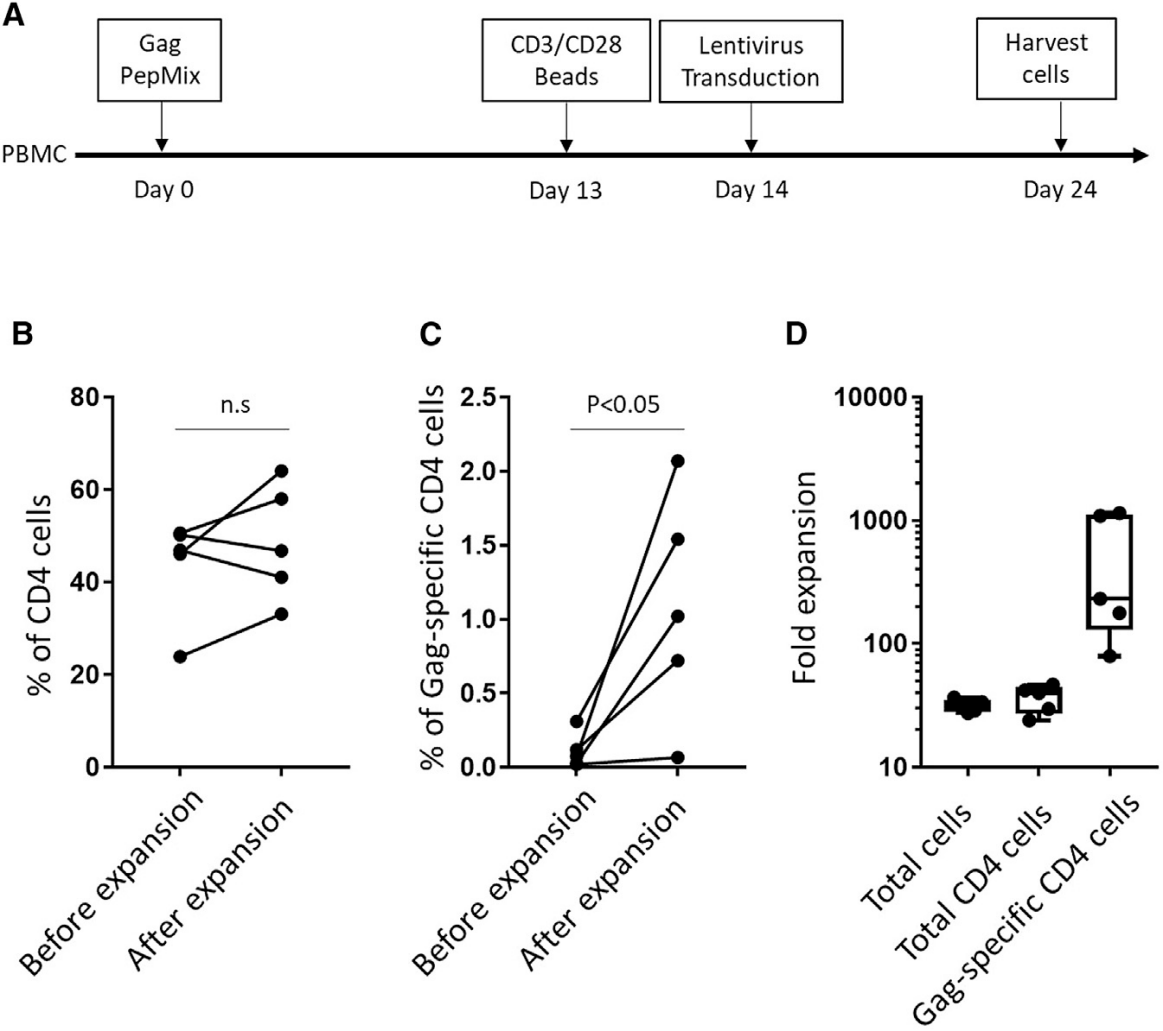
Expansion of HIV Gag-Specific CD4 T Cells by Antigen-Specific Enrichment and Nonspecific Stimulation (A) Experimental design to expand HIV Gag-specific T cells. (B and C) The percentage of CD4 (B) and Gag-specific CD4 T cells (C) before and after expansion. Paired T Tests were performed. All tests were two-tailed and P values of P<0.05 were considered significant. (D) Fold expansion of total cells, total CD4 T cells, and HIV Gag-specific CD4 T cells. Boxplots show all points (minimum [min] to maximum [max]) and mean.

Based on earlier reports,^31^ we started by testing a two-step protocol including enrichment of antigen-specific T cells followed by nonspecific expansion (Figure 2A). PBMC from HIV-positive individuals were stimulated with a pool of peptides representing the HIV Gag protein (JPT GAG PepMix) for 12 days. On day 12, cells were stimulated with CD3/CD28 Dynabeads and transduced with lentivirus and then cultured for an additional 12 days. To prevent possible HIV outgrowth, the protease inhibitor saquinavir was added to the culture medium. HIV protease inhibitor does not affect lentivirus transduction and was reported as improving proliferation of T cells from HIV-infected individuals by a mechanism independent of virus suppression.^32^

The percentages of CD4-positive and Gag-specific T cells were monitored before and after cell expansion. Gag-specific T cells were identified by intracellular staining for interferon (IFN)-γ after peptide restimulation. The expansion of cell subsets was calculated by dividing the total number of cells in the final product with the cell number in the starting material. After expansion, the CD4 T cell percentage was mostly unchanged (Figure 2B), but the percentage of Gag-specific CD4 T cells was increased significantly (from 0.11% ± 0.05% to 1.08% ± 0.34%, mean ± SEM; Figure 2C). Figure 2D shows the relative increases in total cells (31.7 ± 1.645), total CD4 T cells (36.51 ± 4.175), and HIV Gag-specific CD4 T cells (545.4 ± 235.5). Although the Gag-specific CD4 T cells were increased in number, their percentage in the cell product remained low.

To increase the frequency of Gag-specific CD4 T cells in the final product, we tested a physical enrichment method using the Miltenyi Cytokine Capture System (CCS). CCS selection enriched the Gag-specific T cells. However, after nonspecific stimulation and expansion, the percentage of HIV Gag-specific T cells was again very low (Figure S3). It is likely that restimulation with antigen caused activation-induced cell death (AICD) of target cells, but we did not conduct studies to define the mechanism for cell loss.

**Figure 3:**
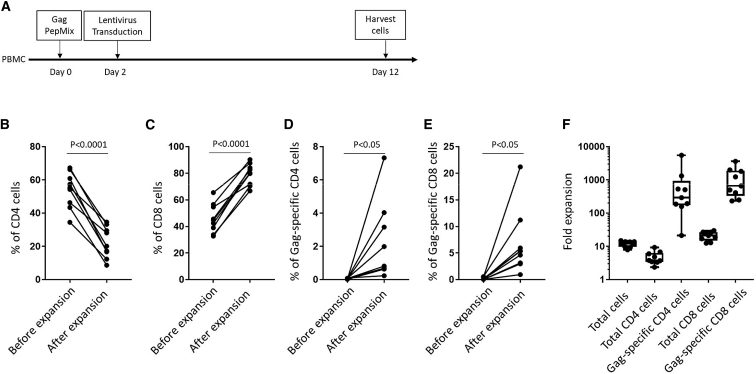
Expansion of HIV Gag-Specific CD4 T Cells by Peptide Stimulation (A) Experimental design to expand HIV Gag-specific T cells. (B, C, D, and E) The percentage of CD4 (B), CD8 (C), Gag-specific CD4 (D), and CD8 T cells (E) before and after expansion. Paired T Tests were performed. All tests were two-tailed and P values of P<0.05 were considered significant. (F) Fold expansion of total cells, total CD4 T cells, HIV Gag-specific CD4 T cells, total CD8 T cells, and HIV Gag-specific CD8 T cells. Boxplots show all points (min to max) and mean.

We next tested whether elimination of the nonspecific expansion step would improve the yield of antigen-specific CD4 T cells (Figure 3A). We observed that CD4 percentages declined (from 53.97% ± 3.61% to 22.26% ± 3.12%; Figure 3B), and CD8 percentages increased (from 46.09% ± 3.64% to 79.3% ± 2.684%; Figure 3C), suggesting overgrowth of the culture by CD8 T cells. Both Gag-specific CD4 (from 0.052% ± 0.01% to 2.169% ± 0.79%; Figure 3D) and CD8 T cells (from 0.165% ± 0.07% to 6.74% ± 2.04%; Figure 3E) were increased significantly. Figure 3F shows expansion of total cells (11.84 ± 0.8), total CD4 T cells (4.833 ± 0.72), Gag-specific CD4 T cells (971.2 ± 583.5), total CD8 T cells (21.3 ± 2.12), and Gag-specific CD8 T cells (1,192 ± 374.3).

**Figure 4:**
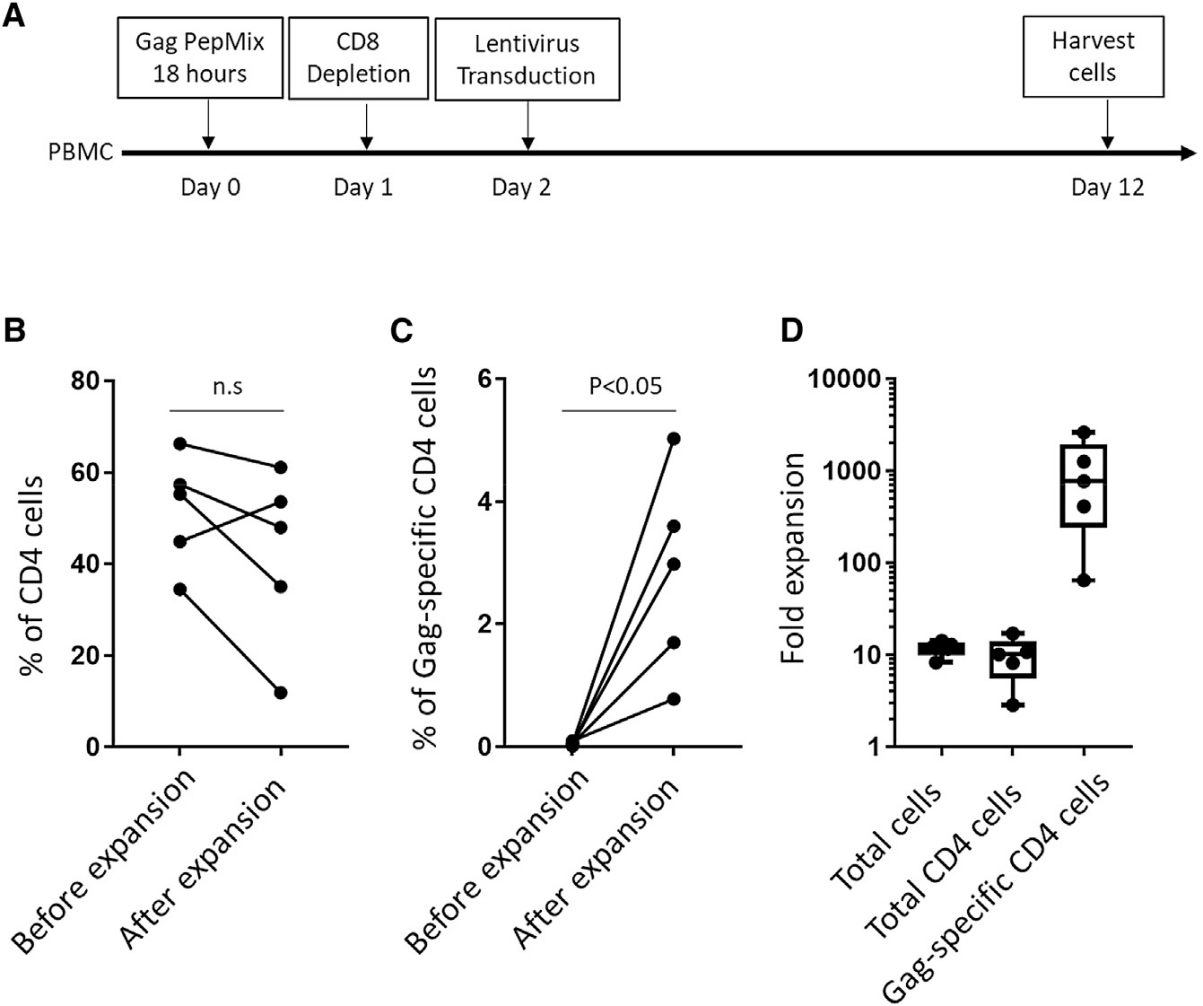
Expansion of HIV Gag-Specific CD4 T Cells by Peptide Stimulation and CD8 T Cell Depletion (A) Experimental design to expand HIV Gag-specific T cells. (B and C) The percentage of CD4 (B) and Gag-specific CD4 T cells (C) before and after expansion. Paired T Tests were performed. All tests were two-tailed and P values of P<0.05 were considered significant. (D) Fold expansion of total cells, total CD4 T cells, and HIV Gag-specific CD4 T cells. Boxplots show all points (min to max) and mean.

To improve the yield of bulk and specific CD4 T cells, CD8 T cells were depleted after the step of peptide stimulation (Figure 4A). This depletion did not correct the CD4 T cell loss, and we still observed reduced CD4 percentages overall (from 51.68% ± 5.48% to 41.94% ± 8.628%; Figure 4B), even though the percentages (from 0.054% ± 0.013% to 2.818% ± 0.74%; Figure 4C) and expansion (1,031 ± 447.3; Figure 4D) of Gag-specific CD4 T cells were improved. To learn why CD4 T cell percentages were reduced after CD8 depletion, we analyzed cell subsets in the product. Surprisingly, we observed high levels of CD3^−^CD56^+^ natural killer (NK) cells (Figure S4A) or Vδ1^+^ γδ T cells (Figure S4B) in several of the cell products, indicating these cell subsets also have growth advantages over CD4 T cells in PBMC from HIV^+^ individuals.

**Figure 5:**
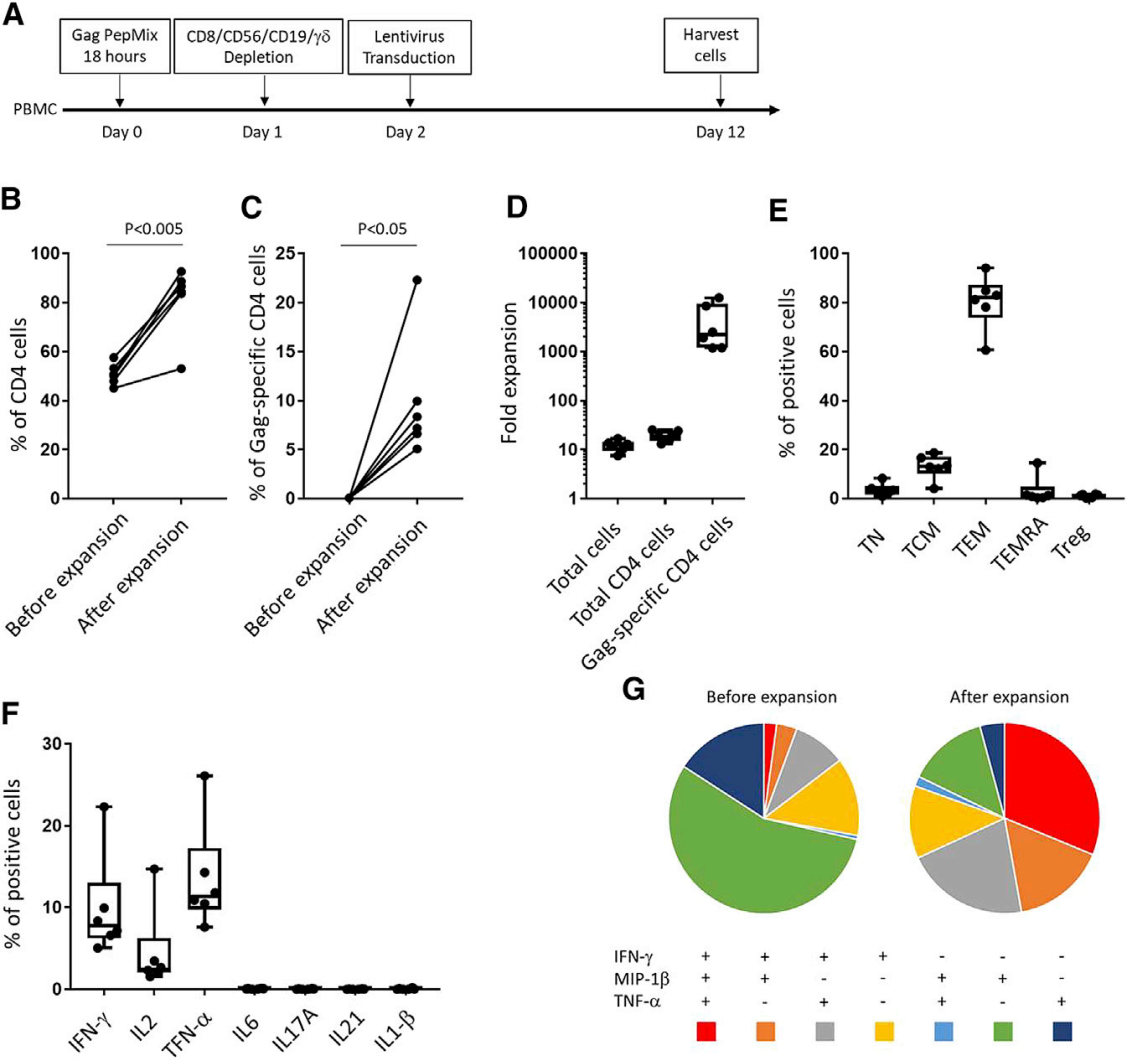
Expansion of HIV Gag-Specific CD4 T Cells by Peptide Stimulation and CD8/CD56/CD19/γδ Depletion (A) Experimental design to expand HIV Gag-specific T cells. (B and C) The percentage of CD4 (B) and Gag-specific CD4 (C) T cells before and after expansion. Paired T Tests were performed. All tests were two-tailed and P values of P<0.05 were considered significant. (D) Fold expansion of total cells, total CD4 T cells, and HIV Gag-specific CD4 T cells. Boxplots show all points (min to max) and mean. (E) The percentage of different T subsets among expanded cells. Boxplots show all points (min to max) and mean. (F) The percentage of CD4 T cells producing different cytokines among expanded cells after restimulation with Gag peptides. Boxplots show all points (min to max) and mean. (G) The pie charts represent the average frequencies of active, cytokine-producing cells, producing every possible combination of the three cytokines analyzed (n = 8; see also Figure S6).

The next step was to deplete additional cell subsets after peptide stimulation, including cells expressing CD8, CD56, γδ T cell receptor, or CD19 (Figure 5A). The expansion of total CD4 and Gag-specific CD4 T cells was improved by the cell-depletion step (Figure S5), resulting in improved yields of total CD4 (from 50.73% ± 1.76% to 81.45% ± 5.84%; Figure 5B) and Gag-specific CD4 (from 0.04% ± 0.01% to 9.91% ± 2.57%) CD4 T cells. Expansion of Gag-specific CD4 T cells (4,607 ± 1,922; Figure 5C) was especially improved by this method. The phenotype of T cells in the product was characterized by expression of CD27 and CD45RA. The major subset in our product was effector memory T cell (TEM) phenotype (80.28% ± 4.51%; Figure 5E). In addition, all products contained central memory T cells (TCMs; 12.99% ± 2.04%; Figure 5E), an immune subset linked to long-term, persisting T cells. Further, CD25^+^FoxP3^+^ regulatory T cell (Treg) levels were very low or undetectable (1.13% ± 0.28%; Figure 5E). In addition to IFN-γ, we measured other cytokines that might be produced by CD4 T cells. After Gag peptide stimulation, the CD4 T cells produced IL-2 and TNF-α but not IL-6, IL-17A, IL-21, or IL-1β. To assess the quality of responding T cells before and after expansion, IFN-γ, TNF-α, and MIP-1β were analyzed simultaneously by multiparameter flow cytometry. The relative proportions of cells making different cytokines are depicted by pie charts representing the mean values among 8 donors (Figures 5G and S6). After expansion, HIV Gag-specific CD4 T cells contained much higher frequencies of multifunctional IFN-γ-, TNF-α-, and MIP-1β-producing cells.

**Figure 6:**
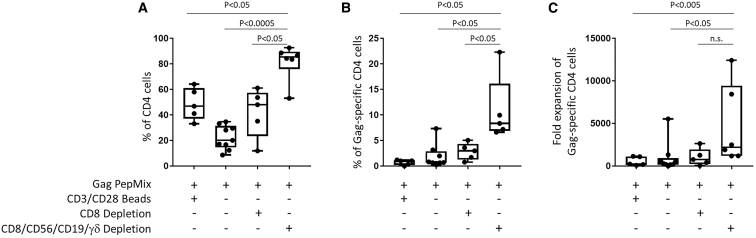
Comparison of the Results of Different Protocols (A and B) The percentage of CD4 (A) and Gag-specific CD4 T cells (B) before and after expansion. (C) Fold expansion of HIV Gag-specific CD4 T cells. Boxplots show all points (min to max) and mean. Mann-Whitney tests were performed. All tests were two-tailed and P values of P<0.05 were considered significant.

After comparing different protocols (Figure 6), we concluded that depletion of CD8^+^, CD56^+^, CD19^+^, and γδ^+^ cell subsets optimized the percentages and yields of Gag-specific CD4 T cells.

### Evaluating Transduction Efficiency in Expanded HIV Gag-Specific CD4 T Cells

**Figure 7:**
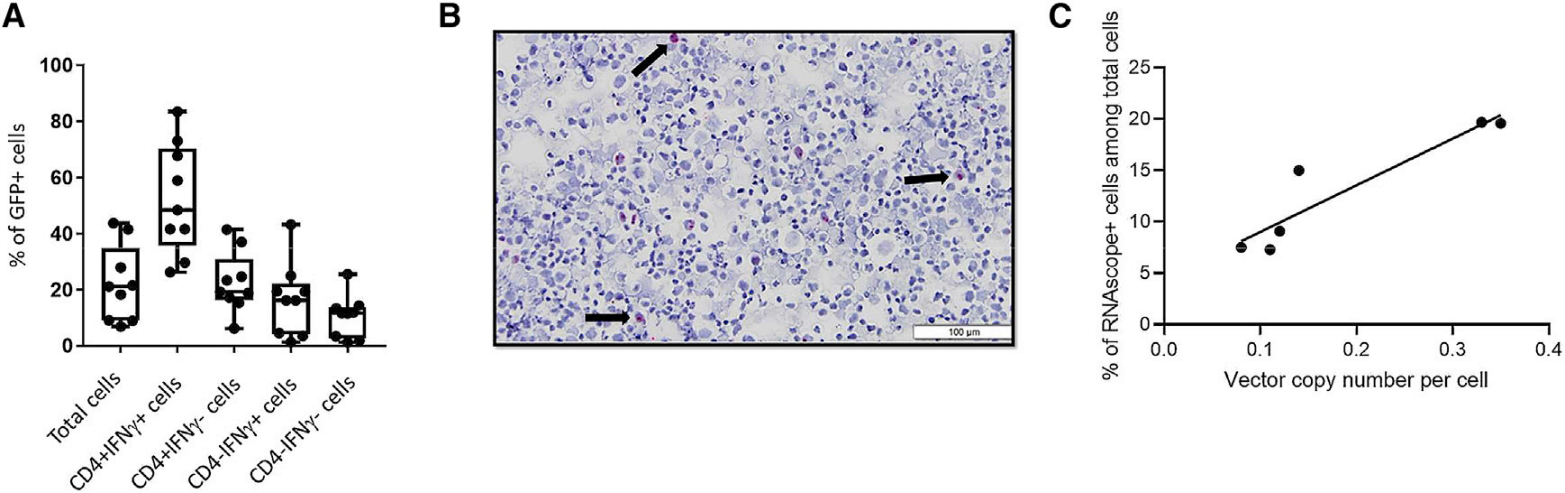
Evaluation of Lentivirus Transduction Efficiency (A) Percentage of lentivirus AGT103-GFP-transduced cells among total cells or different subsets (see also Figure S7). Boxplots show all points (min to max) and mean. (B) A representative image of RNAScope to detect AGT103-transduced cells. (C) Correlation between percentage of transduced cells by RNAScope and average vector copy number per cell.

We optimized the cell process to improve the yield of antigen-specific CD4 T cells and to achieve efficient transduction of specific cells. For initial studies, we used AGT103 lentivirus carrying GFP (AGT103.G) to evaluate transduction efficiency. Because intracellular staining causes significant GFP signal loss, we used cytokine capture assay to detect IFN-γ^+^ antigen-specific CD4 T cells expressing GFP. Although the overall transduction of total cells was modest (22.2% ± 4.52%; Figures 7A and S7A), the IFN-γ-positive, antigen-specific CD4 T cells were transduced at a much higher efficiency (52.36% ± 6.58%; Figures 7A and S7B) compared to other cells. This result is reasonable given that antigen-specific CD4 T cells received TCR stimulation, proliferated faster, and were easier to transduce with lentivirus vector.

The therapeutic vector AGT103 is intended for use in manufacturing the clinical-grade product AGT103-T and does not express a tag or marker (e.g., GFP). Therefore, to evaluate transduction efficiency in cell products, we optimized and qualified an *in situ* hybridization (ISH)-based method (RNAScope; ACD Bio). The method detects RNA transcripts of the woodchuck hepatitis virus post-transcriptional response element (WPRE), which is encoded by AGT103. Positive cells (Figure 7B; showing a representative image) are quantified using image analysis software that determines the frequency of AGT103-transduced cells, which is compared to vector copy number that was measured by qPCR (Figure 7C).

### Developing a Protocol for Good Manufacturing Practice (GMP)-Grade Manufacturing

Our cell process requires PBMC isolation, activation, depletion, transduction, expansion, and cryopreservation steps. For manufacturing of clinical cell products, we implemented the AGT103-T protocol on a CliniMACS Prodigy T Cell Transduction (TCT) system (Miltenyi Biotec, Sunnyvale, CA). The process is semi-automated in a partially closed system for GMP-grade manufacturing. To validate the automated TCT protocol on the CliniMACS Prodigy, large-scale pilot run experiments were performed using apheresis materials from HIV^+^ donors enrolled in the CS-168 specimen collection study (ClinicalTrials.gov: NCT03215004). Because GMP-grade reagents for γδ T cell depletion are not available currently, only CD8/CD56/CD19-positive cells were depleted in the automated cell process.

**Figure 8:**
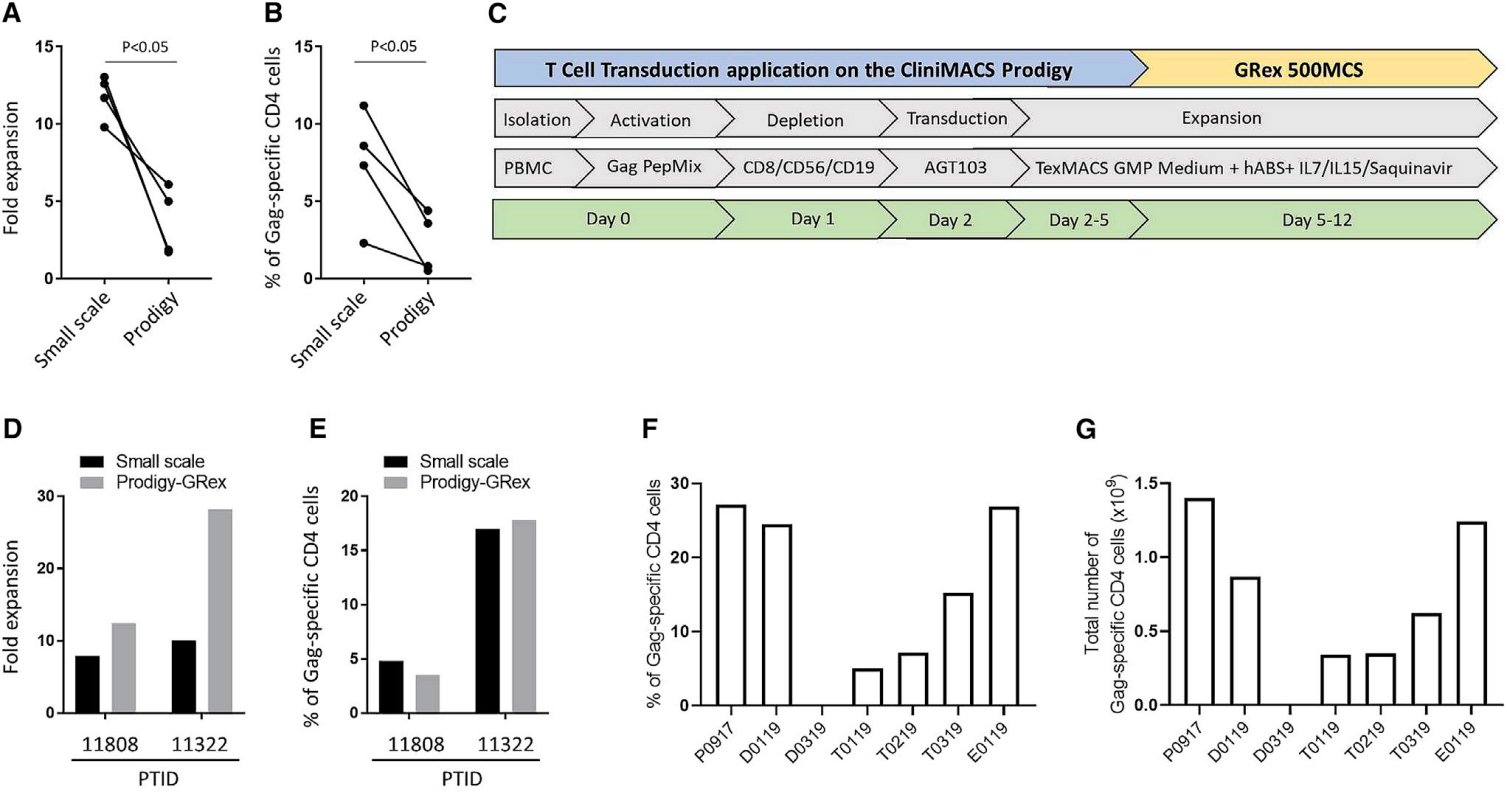
GMP-Grade Manufacturing of HIV Gag-Specific CD4 T Cells (A and B) Comparison of the fold expansion of cells (A) and the percentage of Gag-specific CD4 T cells (B) with small-scale or Prodigy expansion. Paired T Tests were performed. All tests were two-tailed and P values of P<0.05 were considered significant. (C) Experimental design to manufacture HIV Gag-specific, lentivirus-modified CD4 T cells in a clinical-scale and GMP condition. (D and E) Comparison of the fold expansion of cells (D) and the percentage of Gag-specific CD4 T cells (E) with small-scale or Prodigy-G-Rex expansion. (F and G) Percentage (F) and total number (G) of HIV Gag-specific CD4 T cells from 7 donors with the clinical-scale Prodigy-G-Rex expansion protocol.

Unfortunately, with specimens from HIV^+^ patients, cell expansion (Figure 8A) and Gag-specific CD4 T cell percentages (Figure 8B) were lower in the CliniMACS Prodigy compared to small-scale experiments. This might have been due to the volume limitation of a CentriCult Unit (maximum 250 mL) for cell culture and the repeated centrifugation and resuspension steps necessary for daily medium exchanges. To solve this problem, we used the CliniMACS Prodigy in combination with a gas-permeable G-Rex cell-culture device (Figure 8C). PBMC isolation, activation, depletion, and transduction were performed in the CliniMACS Prodigy. After transduction, cells were transferred into a G-Rex 500M-CS vessel for expansion. To simplify the procedure and reduce manufacturing cost, we wondered whether cytokine and saquinavir feeding could be avoided. We tested the stability of IL-7 and IL-15 during cell culture. Both IL-7 and IL-15 are quite stable from day 5 to day 14 (Figure S8). Saquinavir in the medium was also effective for inhibiting HIV replication until the end of the culture period (data not shown). Consequently, after cells were transferred into a G-Rex vessel no additional feedings or cell manipulations were needed until cell harvest on day 12. We performed large-scale pilot runs with two patient samples to validate the protocol. Compared with small-scale experiments, expansion of total cells (Figure 8D) and Gag-specific CD4 T cell percentages (Figure 8E) were improved by incorporating the static culture step using a G-Rex vessel. We also completed large-scale manufacturing with apheresis materials from 7 additional donors and obtained consistent results for 6 of these donors. One donor had a poor response to Gag peptide stimulation. For Gag-specific CD4 T cells, the average percentage and yield in final product were 15.13% (Figure 8F) and 7 × 10^8^ (Figure 8G) respectively.

## Discussion

The destruction of CD4 T cell immunity during chronic HIV infection causes immune system dysfunction and failure to control HIV. Recent studies in HIV controllers highlighted the importance for HIV-specific CD4 T cells in controlling HIV replication and disease progression;^18^^,^^24^^,^^26^, ^27^, ^28^ this prompted us to design a cell and gene therapy to reconstitute these critical CD4 T cells. Adoptive, autologous, antigen-specific CD4 T cell therapy is a promising strategy to rebuild an effective immune system against HIV that might improve clinical status and reduce dependence on antiretroviral therapy. However, the unique characteristics of HIV infection created barriers to expand HIV-specific CD4 T cells and has prevented, until now, the development of cell and gene therapies with cells enriched for the HIV-specific CD4 T cell subset. We developed an optimized protocol for efficient clinical-scale manufacturing of highly enriched, HIV-specific CD4 T cells that makes application of this method feasible for treating HIV infection. The combination of cell enrichment with the ability to protect these cells against HIV-mediated depletion has achieved a new type of immunotherapy for HIV disease.

Although HIV-specific CD4 T cells in peripheral blood can be detected in most, if not all, HIV-infected individuals, their frequencies are extremely low.^33^^,^^34^ Moreover, HIV-specific CD4 T cells that evade depletion often show functional impairment,^35^ making it difficult to generate a cell product containing sufficient frequencies and numbers of these cells. Previous studies for expanding tumor antigen-specific T cells used a complex, combined method of *in vivo* priming, *ex vivo* antigen-specific enrichment, and nonspecific expansion.^31^ We tested this method for expanding HIV-specific T cells but were not successful. Instead, we developed a simpler protocol that expands HIV-specific CD4 T cells with higher efficiency and lower cost and requires only 12 days. During cell-process development, we observed surprising overgrowth of CD8, NK, and even Vδ1 T cells in some cultures. This might be unique to HIV infection due to overactivation of these subsets and exhaustion of CD4 T cells during chronic infection.^36^, ^37^, ^38^, ^39^, ^40^, ^41^ We did not observe this phenomenon when expanding cytomegalovirus (CMV)-specific T cells from healthy donors (not shown). The depletion of CD8, NK, and γδ T cells after peptide stimulation significantly enhanced HIV-specific CD4 T cell expansion. Interestingly, when B cells were depleted at the same time, the results were improved. We chose to delete these subsets subsequent to the peptide stimulation to minimize any impact on the efficiency of T cell responses to peptides.

The modification of CD4 T cells for HIV resistance may help them persist *in vivo*. Our lentivirus vector (AGT103) targets CCR5 plus HIV Tat/Vif and is efficient for blocking both R5- and X4-tropic HIV infection and/or replication. Importantly, AGT103 transduction also inhibits virus release from latently infected cells. AGT103 is a third-generation, self-inactivating lentivirus vector carrying three inhibitory RNAs that are embedded into an endogenous miRNA backbone. Several clinical trials, including chimeric antigen receptor T cell (CAR-T) therapy, have shown that lentivirus-modified cells are safe for infusion.^42^ Analysis of lentiviral vector integration in HIV^+^ study subjects receiving infusions of autologous, gene-modified CD4 T cells provided no evidence for abnormal expansion of cells due to vector-mediated, insertional activation of proto-oncogenes.^43^

The manufacturing of genetically modified T cells, especially from persons with HIV, is complex. The CliniMACS Prodigy automates several steps from PBMC purification, enrichment, activation, transduction, and expansion to final formulation and sampling in a closed sterile, single-use tubing set.^44^, ^45^, ^46^ However, manufacturing using only the CliniMACS Prodigy lead to low yields of antigen-specific and total CD4 T cells compared to small-scale experiments. The limited volume (250 mL) of the Prodigy’s CentriCult-Unit caused us to maintain very high cell concentrations (>1 × 10^7^/mL). At these cell concentrations, proliferation of antigen-specific CD4 T cells may be inhibited, and the frequent centrifugation needed for daily medium replacement seemed to cause death of activated cells, particularly the antigen-stimulated cell subset. When we transferred cells at day 5 from the CliniMACS Prodigy to a G-Rex container for static cell culture, expansion of total and HIV-specific CD4 T cells were improved.

In this study, we focused on expanding HIV Gag-specific CD4 T cells because of their importance for viral suppression and improved clinical outcomes. Induction of Gag-specific CD4 T cell responses during acute HIV infection is associated with improved viral control.^47^ Antigen-specific CD4 T cells in HIV controllers preferentially target epitopes in the Gag protein compared with other viral proteins, whereas noncontrollers exhibited a dominant targeting of epitopes in the envelope protein.^34^ Robust and sustained CD4 T cell proliferative responses to the Gag p24 antigen correlated with control of viremia and lack of disease progression after long-term transfusion-acquired HIV-1 infection.^48^ The HIV-specific cytotoxic CD4 T cells also contribute to viral suppression,^26^, ^27^, ^28^^,^^49^ and HIV-specific cytotoxic CD4 T cells in our product (Figure S9) may provide an enhanced effect to reduce the burden of infected cells. In the future, we may incorporate stimulation for other HIV antigens, besides Gag, to broaden and deepen the HIV-specific CD4 T cell population. The optimized cell manufacturing protocol described here may also be used to expand other virus or cancer antigen-specific CD4 T cells to accelerate clinical application of this promising therapeutic strategy that combines CD4 T cell enrichment with a potent lentivirus vector to create a unique immunotherapy for HIV disease.

## Materials and Methods

### GMP-Grade Manufacturing in the CliniMACS Prodigy and G-Rex

The semi-automated TCT system was performed on the CliniMACS Prodigy using the Tubing Set TS520 and the TCT process. Unless noted elsewhere, reagents and materials were obtained from Miltenyi Biotec (Bergisch Gladbach, Germany). An overview of the general procedure is depicted in Figure 8C. In brief, apheresis materials from HIV^+^ donors were processed on a CliniMACS Prodigy device by running an internally developed customized application (CAP): A001_Apheresis_Separation, with a TS520 tubing set. The program executes a Ficoll (GE Healthcare) separation to reduce red blood cells (RBCs) and granulocytes. After Ficoll, the cells were sampled and analyzed for cell count, viability, and phenotype. Dry cell pellet samples were retained for additional testing. The remaining post-Ficoll cells were returned to the same CliniMACS Prodigy device for activation, transduction, and cultivation. The T Cell Transduction process program was executed on the same CliniMACS Prodigy device with the same TS520 tubing set, according to the TCT User Manual, with cultivation of the target population as the major goal. On day 0, the post-Ficoll cells were resuspended in 100 mL of culture media and brought into the CentriCult Unit (CCU) of the TS520 tubing set before incubation with PepMix HIV-1 (GAG) Ultra (1 μg/mL; JPT, Berlin, Germany) for ≥16 h at 37°C in static culture. Culture media were TexMACS GMP medium with 3% human AB serum (Valley Medical), 12.5 ng/mL IL-7, 12.5 ng/mL IL-15, and 100 nM saquinavir (Sigma, Burlington, MA, USA). A sample of the culture media was analyzed for baseline metabolite levels. On day 1, after a 16-h incubation, the TCT process was stopped, and the culture was sampled and analyzed for cell count, viability, phenotype, and intracellular IFN-γ peptide stimulation prior to CD8/19/56 cell depletion. Culture supernatant was assessed for metabolite levels. A CD8/19/56 depletion was performed using another internally developed CAP program: A001_Depletion. Postdepletion, the cells were resuspended in 70 mL of culture media and placed in static culture. On day 2, the seeded cells were sampled and analyzed for cell count, viability, and phenotype. Culture supernatant was assessed for metabolite levels. The remaining seeded cells were transduced with the AGT103-ER lentiviral vector (Lentigen, Gaithersburg, MD, USA) at a MOI of 5 in approximately 100 mL of culture media. On day 3, a feed of 100 mL was added to suspend the cells in 200 mL of culture media. On day 5, the seeded cells were sampled and analyzed for cell count, viability, and phenotype. Dry cell pellet samples were retained for additional testing. Culture supernatant was assessed for metabolite levels. The remaining seeded cells were harvested and distributed into G-Rex culture devices at the equivalent seeding density of 1 × 10^6^ cells/cm^2^ in G-Rex 500M-CS (Wilson Wolf, St. Paul, MN, USA). On day 12, the G-Rex 500M-CS was harvested and analyzed for cell count, viability, and phenotype. Peptide pulse and intracellular IFN- γ staining were performed to measure HIV-specific CD4 T cells. Dry cell pellet samples were retained for additional testing. Culture supernatant was assessed for metabolites, cytokine levels, P24, and mycoplasma. The cells were formulated in cryopreservation media. Samples were taken for sterility and endotoxin, and then the remaining cells were cryopreserved.

### PBMC Isolation and Cell Culture

Whole blood was obtained from HIV-infected adult volunteers who provided written, informed consent to participate in the CS-168 specimen collection study (NCT03215004). The protocol was approved by the Institutional Review Board of Providence Hospital, Washington, DC NW. Total lymphocytes were separated from EDTA-anticoagulated peripheral blood by density gradient centrifugation (Ficoll-Paque; Amersham Biosciences, Little Chalfont, UK). PBMC were cultured in RPMI 1640, supplemented with 10% fetal bovine serum (FBS; Gibco, Gaithersburg, MD, USA), 2 mM l-glutamine, and penicillin-streptomycin (100 U/mL and 100 mg/mL, respectively). 293T, JC53 cells were cultured in DMEM, supplemented with 10% FBS, 2 mM l-glutamine and penicillin-streptomycin (100 U/mL and 100 mg/mL, respectively). J1.1 and C8166 cells were cultured in RPMI 1640, supplemented with 10% FBS, 2 mM l-glutamine, and penicillin-streptomycin (100 U/mL and 100 mg/mL, respectively). To induce HIV replication, J1.1 cells were treated with 50 ng/mL TNF-α (R&D Systems, Minneapolis, MN, USA).

### Small-Scale Expansion of HIV Gag-Specific CD4 T Cells

Culture media in these experiments were TexMACS GMP medium with 3% human AB serum (Valley Medical, Purcellville, VA, USA), 12.5 ng/mL IL-7, 12.5 ng/mL IL-15, and 100 nM saquinavir (Sigma, Burlington, MA, USA). For the expansion to include antigen-specific enrichment and nonspecific expansion, 2 × 10^6^ PBMC from vaccinated patients was first stimulated with the HIV Gag peptide pool (1 μg/mL individual peptide, GAG PepMix; JPT, Berlin, Germany) or DMSO (negative control) in culture media for 12 days for an antigen-specific T cell enrichment, or HIV-specific T cells were enriched by a Cytokine Capture System. On day 12, cells were stimulated with CD3/CD28 beads (Thermo Fisher Scientific, Waltham, MA, USA) and transduced with lentivirus AGT103 at MOI 5. Cells were cultured for another 12 days. The number of the cells was evaluated every 2 days, and the cells were diluted to 0.5 × 10^6^/mL with fresh culture media. For the expansion with peptide stimulation and cell depletion, PBMCs (1 × 10^7^) were stimulated with an HIV Gag peptide pool in 1 mL medium in a 24-well plate for 18 h. CD8 (clone SK1)-, γδ (clone B1)-, CD56 (clone 5.1H11)-, or CD19 (clone 4G7)-positive cells were depleted with phycoerythrin (PE)-labeled, specific antibodies (BioLegend, San Diego, CA, USA) and anti-PE microbeads (Miltenyi Biotech, Bergisch Gladbach, Germany). The negatively selected cells were cultured at 2 × 10^6^/mL in culture media. Lentivirus AGT103 was added 24 h later at MOI 5. Fresh culture media were added every 2–3 days during the expansion. At day 14, cells were collected for analysis.

### Flow Cytometry

Unless noted, cells were stained with fluorophore-conjugated monoclonal antibodies (mAb) from BioLegend (San Diego, CA, USA). For cell-surface staining, cells were washed and resuspended in 50–100 μL of RPMI 1640 and then stained with mouse anti-human CD4 clone OKT4, mouse anti-human CD3 clone OKT3, mouse anti-human CD8 clone SK1, mouse anti human CD45RA clone HI100, mouse anti-human CD27 clone MT-271, or mouse anti-human CCR5 clone J418F1, including corresponding isotype controls. For detection of intracellular cytokines, cells were pulsed with 1 μg/mL HIV Gag peptides (PepMix HIV-1 [GAG] Ultra; JPT, Berlin, Germany) and GolgiPlug (BD Biosciences, San Jose, CA, USA) for 4 h; stained with mouse anti-human CD4 clone OKT4 and mouse anti-human CD8 clone SK1; and then fixed, permeabilized, and incubated for 45 min at 4°C with mouse anti-human IFN-γ clone B27, mouse anti-human TNF-α clone mAb11, rat anti-human IL-2 clone MQ1-17H12, rat anti-human IL-6 clone MQ2-13A5, mouse anti-human IL-17A clone BL168, mouse anti-human IL-21 clone 3A3-N2, or mouse anti-human IL-1β clone JK1B-1, including corresponding isotype controls. Intracellular staining solutions were obtained from the Cytofix/Cytoperm Kit (BD Biosciences, San Jose, CA, USA). Cells were washed with staining buffer and resuspended. For detection of FoxP3, cells were stained with mouse anti-human CD4 clone OKT4 and mouse anti-human CD25 clone BC96 and performed FoxP3 staining with FOXP3 Fixation/Permeabilization Buffer. Data were acquired for at least 1 × 10^6^ lymphocytes (gated based on the forward- and side-scatter profiles) from each sample, using a FACSCalibur flow cytometer (BD Biosciences, San Jose, CA, USA). All samples were analyzed using FlowJo software (FlowJo 8.8.2; Tree Star, San Carlos, CA, USA).

### Immunoblot Analysis

Cells were lysed in gel loading buffer (Invitrogen, Carlsbad, CA, USA); samples were boiled for 10 min, and proteins were separated by SDS-PAGE. Proteins were transferred to polyvinylidene fluoride (PVDF) membranes (MilliporeSigma, Burlington, MA, USA) and probed with the antibodies anti-Vif (Aids Reagent Program; catalog number [cat. no.] 6459) and β-actin (MilliporeSigma; cat. no. A1978). An anti-mouse secondary antibody conjugated with horseradish peroxidase (HRP; Bio-Rad, Hercules, CA, USA) was visualized with the Immobilon Western HRP substrate (MilliporeSigma, Burlington, MA, USA) and detected with the Li-Cor C-DiGit Blot Scanner (Lincoln, NE, USA).

### RNAScope Assay

Cryopreserved AGT103-T final product cells were thawed in RPMI medium (Thermo Fisher Scientific) containing 10% FBS and 0.5 mg/mL DNase I (MilliporeSigma, Burlington, MA, USA). Cells were washed once in thawing medium and allowed to rest in a cell-culture incubator for 1 h in RPMI containing 5% human AB serum (MilliporeSigma, Burlington, MA, USA) and 10 ng/mL IL-7 and IL-15 (BioLegend, San Diego, CA, USA). The cells were then stimulated with 10 ng/mL IL-2 (BioLegend) and 20 μL (per 5 × 10^6^ cells) TransAct (Miltenyi Biotec, Bergisch Gladbach, Germany) and incubated for another 3 h. The cells were pooled, centrifuged at 500 × *g* for 5 min, and then resuspended in PBS + 5% FBS (5 × 10^6^ cells per mL). Slides were prepared by loading 200 μL of cell suspension into a cytospin funnel (VWR International, Radnor, PA, USA) and centrifuged using a Cytospin 4 (Thermo Fisher Scientific, Waltham, MA, USA) to create cell spots with approximately 10^6^ cells per spot. Slides were processed as described in the ACD manual for processing of suspension cells and use of RNAScope kits. For work described in this manuscript, the RNAScope 2.5 HD Assay-Red kit was used. The RNAScope probe for WPRE was used to detect AGT103 RNA (cat. no. 450261). Brightfield images (20 fields per cell spot) were obtained using a 40× object (Zeiss) and quantified using ImageJ software (https://www.nih.gov/).

### Statistical Analysis

Paired t test and Mann-Whitney tests were performed using GraphPad Prism version 8.2.1. All tests were two tailed, and p values of <0.05 were considered significant.

## Author Contributions

H.L., T.L., L.X., N.M., and J.B. designed and conducted experiments. T.-W.C. and C.D.P. evaluated experimental data and reviewed the manuscript. C.D.P. and H.L. designed the study and wrote the paper. H.L. designed and performed the experiments for expanding HIV-specific T cells and analyzed the data. T.L. and L.X. designed and generated the lentivirus vector. N.M. performed RNAScope studies and analyzed the data. J.B. and T.-W.C. performed the multi-cytokine experiments and analyzed the data. All authors read, reviewed, and approved the manuscript.

## Conflicts of Interest

H.L., T.L., L.X., N.M., and C.D.P. are employees of and shareholders in American Gene Technologies Inc. (AGT) in Rockville, Maryland. H.L., T.L., L.X., and C.D.P. are inventors of the cell-processing technology and lentivirus vector, and issued patents have been assigned to AGT. J.B. and T.-W.C. are employees of the US National Institutes of Health in Bethesda, Maryland, and received no financial benefit or incentives from AGT and have no conflict of interest.

## Supplemental Information

Document S1. Figures S1–S9

Document S2. Article plus Supplemental Information

## References

[1] Aubert R.D., Kamphorst A.O., Sarkar S., Vezys V., Ha S.J., Barber D.L., Ye L., Sharpe A.H., Freeman G.J., Ahmed R. (2011). Antigen-specific CD4 T-cell help rescues exhausted CD8 T cells during chronic viral infection. Proc. Natl. Acad. Sci. USA.

[2] Heslop H.E., Ng C.Y., Li C., Smith C.A., Loftin S.K., Krance R.A., Brenner M.K., Rooney C.M. (1996). Long-term restoration of immunity against Epstein-Barr virus infection by adoptive transfer of gene-modified virus-specific T lymphocytes. Nat. Med..

[3] Hinrichs C.S., Rosenberg S.A. (2014). Exploiting the curative potential of adoptive T-cell therapy for cancer. Immunol. Rev..

[4] Kamphorst A.O., Ahmed R. (2013). CD4 T-cell immunotherapy for chronic viral infections and cancer. Immunotherapy.

[5] Leen A.M., Myers G.D., Sili U., Huls M.H., Weiss H., Leung K.S., Carrum G., Krance R.A., Chang C.C., Molldrem J.J. (2006). Monoculture-derived T lymphocytes specific for multiple viruses expand and produce clinically relevant effects in immunocompromised individuals. Nat. Med..

[6] Muranski P., Restifo N.P. (2009). Adoptive immunotherapy of cancer using CD4(+) T cells. Curr. Opin. Immunol..

[7] Saglio F., Hanley P.J., Bollard C.M. (2014). The time is now: moving toward virus-specific T cells after allogeneic hematopoietic stem cell transplantation as the standard of care. Cytotherapy.

[8] Patel S., Jones R.B., Nixon D.F., Bollard C.M. (2016). T-cell therapies for HIV: Preclinical successes and current clinical strategies. Cytotherapy.

[9] Bollard C.M., Gottschalk S., Huls M.H., Molldrem J., Przepiorka D., Rooney C.M., Heslop H.E. (2006). In vivo expansion of LMP 1- and 2-specific T-cells in a patient who received donor-derived EBV-specific T-cells after allogeneic stem cell transplantation. Leuk. Lymphoma.

[10] Bollard C.M., Gottschalk S., Torrano V., Diouf O., Ku S., Hazrat Y., Carrum G., Ramos C., Fayad L., Shpall E.J. (2014). Sustained complete responses in patients with lymphoma receiving autologous cytotoxic T lymphocytes targeting Epstein-Barr virus latent membrane proteins. J. Clin. Oncol..

[11] Leen A.M., Bollard C.M., Mendizabal A.M., Shpall E.J., Szabolcs P., Antin J.H., Kapoor N., Pai S.Y., Rowley S.D., Kebriaei P. (2013). Multicenter study of banked third-party virus-specific T cells to treat severe viral infections after hematopoietic stem cell transplantation. Blood.

[12] Leen A.M., Christin A., Myers G.D., Liu H., Cruz C.R., Hanley P.J., Kennedy-Nasser A.A., Leung K.S., Gee A.P., Krance R.A. (2009). Cytotoxic T lymphocyte therapy with donor T cells prevents and treats adenovirus and Epstein-Barr virus infections after haploidentical and matched unrelated stem cell transplantation. Blood.

[13] Chakrabarti S., Mautner V., Osman H., Collingham K.E., Fegan C.D., Klapper P.E., Moss P.A., Milligan D.W. (2002). Adenovirus infections following allogeneic stem cell transplantation: incidence and outcome in relation to graft manipulation, immunosuppression, and immune recovery. Blood.

[14] Walls T., Shankar A.G., Shingadia D. (2003). Adenovirus: an increasingly important pathogen in paediatric bone marrow transplant patients. Lancet Infect. Dis..

[15] Feuchtinger T., Matthes-Martin S., Richard C., Lion T., Fuhrer M., Hamprecht K., Handgretinger R., Peters C., Schuster F.R., Beck R. (2006). Safe adoptive transfer of virus-specific T-cell immunity for the treatment of systemic adenovirus infection after allogeneic stem cell transplantation. Br. J. Haematol..

[16] Zandvliet M.L., Falkenburg J.H., van Liempt E., Veltrop-Duits L.A., Lankester A.C., Kalpoe J.S., Kester M.G., van der Steen D.M., van Tol M.J., Willemze R. (2010). Combined CD8+ and CD4+ adenovirus hexon-specific T cells associated with viral clearance after stem cell transplantation as treatment for adenovirus infection. Haematologica.

[17] Lichterfeld M., Kaufmann D.E., Yu X.G., Mui S.K., Addo M.M., Johnston M.N., Cohen D., Robbins G.K., Pae E., Alter G. (2004). Loss of HIV-1-specific CD8+ T cell proliferation after acute HIV-1 infection and restoration by vaccine-induced HIV-1-specific CD4+ T cells. J. Exp. Med..

[18] Rosenberg E.S., Billingsley J.M., Caliendo A.M., Boswell S.L., Sax P.E., Kalams S.A., Walker B.D. (1997). Vigorous HIV-1-specific CD4+ T cell responses associated with control of viremia. Science.

[19] Sant A.J., McMichael A. (2012). Revealing the role of CD4(+) T cells in viral immunity. J. Exp. Med..

[20] An D.S., Donahue R.E., Kamata M., Poon B., Metzger M., Mao S.H., Bonifacino A., Krouse A.E., Darlix J.L., Baltimore D. (2007). Stable reduction of CCR5 by RNAi through hematopoietic stem cell transplant in non-human primates. Proc. Natl. Acad. Sci. USA.

[21] Cannon P., June C. (2011). Chemokine receptor 5 knockout strategies. Curr. Opin. HIV AIDS.

[22] Tebas P., Stein D., Tang W.W., Frank I., Wang S.Q., Lee G., Spratt S.K., Surosky R.T., Giedlin M.A., Nichol G. (2014). Gene editing of CCR5 in autologous CD4 T cells of persons infected with HIV. N. Engl. J. Med..

[23] van Lunzen J., Glaunsinger T., Stahmer I., von Baehr V., Baum C., Schilz A., Kuehlcke K., Naundorf S., Martinius H., Hermann F. (2007). Transfer of autologous gene-modified T cells in HIV-infected patients with advanced immunodeficiency and drug-resistant virus. Mol. Ther..

[24] Gloster S.E., Newton P., Cornforth D., Lifson J.D., Williams I., Shaw G.M., Borrow P. (2004). Association of strong virus-specific CD4 T cell responses with efficient natural control of primary HIV-1 infection. AIDS.

[25] Ferrando-Martínez S., Casazza J.P., Leal M., Machmach K., Muñoz-Fernández M.Á., Viciana P., Koup R.A., Ruiz-Mateos E. (2012). Differential Gag-specific polyfunctional T cell maturation patterns in HIV-1 elite controllers. J. Virol..

[26] Chen H., Li C., Huang J., Cung T., Seiss K., Beamon J., Carrington M.F., Porter L.C., Burke P.S., Yang Y. (2011). CD4+ T cells from elite controllers resist HIV-1 infection by selective upregulation of p21. J. Clin. Invest..

[27] Vigneault F., Woods M., Buzon M.J., Li C., Pereyra F., Crosby S.D., Rychert J., Church G., Martinez-Picado J., Rosenberg E.S. (2011). Transcriptional profiling of CD4 T cells identifies distinct subgroups of HIV-1 elite controllers. J. Virol..

[28] Yang Y., Al-Mozaini M., Buzon M.J., Beamon J., Ferrando-Martinez S., Ruiz-Mateos E., Rosenberg E.S., Pereyra F., Yu X.G., Lichterfeld M. (2012). CD4 T-cell regeneration in HIV-1 elite controllers. AIDS.

[29] Platt E.J., Wehrly K., Kuhmann S.E., Chesebro B., Kabat D. (1998). Effects of CCR5 and CD4 cell surface concentrations on infections by macrophagetropic isolates of human immunodeficiency virus type 1. J. Virol..

[30] Perez V.L., Rowe T., Justement J.S., Butera S.T., June C.H., Folks T.M. (1991). An HIV-1-infected T cell clone defective in IL-2 production and Ca2+ mobilization after CD3 stimulation. J. Immunol..

[31] Dang Y., Knutson K.L., Goodell V., dela Rosa C., Salazar L.G., Higgins D., Childs J., Disis M.L. (2007). Tumor antigen-specific T-cell expansion is greatly facilitated by in vivo priming. Clin. Cancer Res..

[32] Lu W., Andrieu J.M. (2000). HIV protease inhibitors restore impaired T-cell proliferative response in vivo and in vitro: a viral-suppression-independent mechanism. Blood.

[33] Migueles S.A., Tilton J.C., Connors M. (2004). Advances in understanding immunologic control of HIV infection. Curr. HIV/AIDS Rep..

[34] Ranasinghe S., Flanders M., Cutler S., Soghoian D.Z., Ghebremichael M., Davis I., Lindqvist M., Pereyra F., Walker B.D., Heckerman D., Streeck H. (2012). HIV-specific CD4 T cell responses to different viral proteins have discordant associations with viral load and clinical outcome. J. Virol..

[35] Porichis F., Kaufmann D.E. (2011). HIV-specific CD4 T cells and immune control of viral replication. Curr. Opin. HIV AIDS.

[36] De Paoli P., Gennari D., Martelli P., Basaglia G., Crovatto M., Battistin S., Santini G. (1991). A subset of gamma delta lymphocytes is increased during HIV-1 infection. Clin. Exp. Immunol..

[37] Giorgi J.V., Ho H.N., Hirji K., Chou C.C., Hultin L.E., O’Rourke S., Park L., Margolick J.B., Ferbas J., Phair J.P., The Multicenter AIDS Cohort Study Group (1994). CD8+ lymphocyte activation at human immunodeficiency virus type 1 seroconversion: development of HLA-DR+ CD38- CD8+ cells is associated with subsequent stable CD4+ cell levels. J. Infect. Dis..

[38] Giorgi J.V., Hultin L.E., McKeating J.A., Johnson T.D., Owens B., Jacobson L.P., Shih R., Lewis J., Wiley D.J., Phair J.P. (1999). Shorter survival in advanced human immunodeficiency virus type 1 infection is more closely associated with T lymphocyte activation than with plasma virus burden or virus chemokine coreceptor usage. J. Infect. Dis..

[39] Naranbhai V., Altfeld M., Karim S.S., Ndung’u T., Karim Q.A., Carr W.H. (2013). Changes in Natural Killer cell activation and function during primary HIV-1 Infection. PLoS ONE.

[40] Pauza C.D., Poonia B., Li H., Cairo C., Chaudhry S. (2015). γδ T Cells in HIV Disease: Past, Present, and Future. Front. Immunol..

[41] Sereti I., Altfeld M. (2016). Immune activation and HIV: an enduring relationship. Curr. Opin. HIV AIDS.

[42] Milone M.C., O’Doherty U. (2018). Clinical use of lentiviral vectors. Leukemia.

[43] Wang G.P., Levine B.L., Binder G.K., Berry C.C., Malani N., McGarrity G., Tebas P., June C.H., Bushman F.D. (2009). Analysis of lentiviral vector integration in HIV+ study subjects receiving autologous infusions of gene modified CD4+ T cells. Mol. Ther..

[44] Fernández L., Fernández A., Mirones I., Escudero A., Cardoso L., Vela M., Lanzarot D., de Paz R., Leivas A., Gallardo M. (2019). GMP-Compliant Manufacturing of NKG2D CAR Memory T Cells Using CliniMACS Prodigy. Front. Immunol..

[45] Mock U., Nickolay L., Philip B., Cheung G.W., Zhan H., Johnston I.C.D., Kaiser A.D., Peggs K., Pule M., Thrasher A.J., Qasim W. (2016). Automated manufacturing of chimeric antigen receptor T cells for adoptive immunotherapy using CliniMACS prodigy. Cytotherapy.

[46] Zhang W., Jordan K.R., Schulte B., Purev E. (2018). Characterization of clinical grade CD19 chimeric antigen receptor T cells produced using automated CliniMACS Prodigy system. Drug Des. Devel. Ther..

[47] Schieffer M., Jessen H.K., Oster A.F., Pissani F., Soghoian D.Z., Lu R., Jessen A.B., Zedlack C., Schultz B.T., Davis I. (2014). Induction of Gag-specific CD4 T cell responses during acute HIV infection is associated with improved viral control. J. Virol..

[48] Dyer W.B., Zaunders J.J., Yuan F.F., Wang B., Learmont J.C., Geczy A.F., Saksena N.K., McPhee D.A., Gorry P.R., Sullivan J.S. (2008). Mechanisms of HIV non-progression; robust and sustained CD4+ T-cell proliferative responses to p24 antigen correlate with control of viraemia and lack of disease progression after long-term transfusion-acquired HIV-1 infection. Retrovirology.

[49] Soghoian D.Z., Jessen H., Flanders M., Sierra-Davidson K., Cutler S., Pertel T., Ranasinghe S., Lindqvist M., Davis I., Lane K. (2012). HIV-specific cytolytic CD4 T cell responses during acute HIV infection predict disease outcome. Sci. Transl. Med..

