# Supplementary material for: Preclinical Development and Clinical-Scale Manufacturing of HIV Gag-Specific, LentivirusModified CD4 T Cells for HIV Functional Cure
Source: Mol Ther Methods Clin Dev. 2020 May 3;17:1048–60. doi: 10.1016/j.omtm.2020.04.024 (PMC7240062; doi:10.1016/j.omtm.2020.04.024)
Supplement: Document S1. Figures S1–S9 [file mmc1.pdf]

OMTM, Volume *17*

Supplemental Information

Preclinical Development and Clinical-Scale Manufacturing of HIV Gag-Specific, Lentivirus Modified CD4 T Cells for HIV Functional Cure

Haishan Li, Tyler Lahusen, Lingzhi Xiao, Nidal Muvarak, Jana Blazkova, Tae-Wook Chun, and C. David Pauza

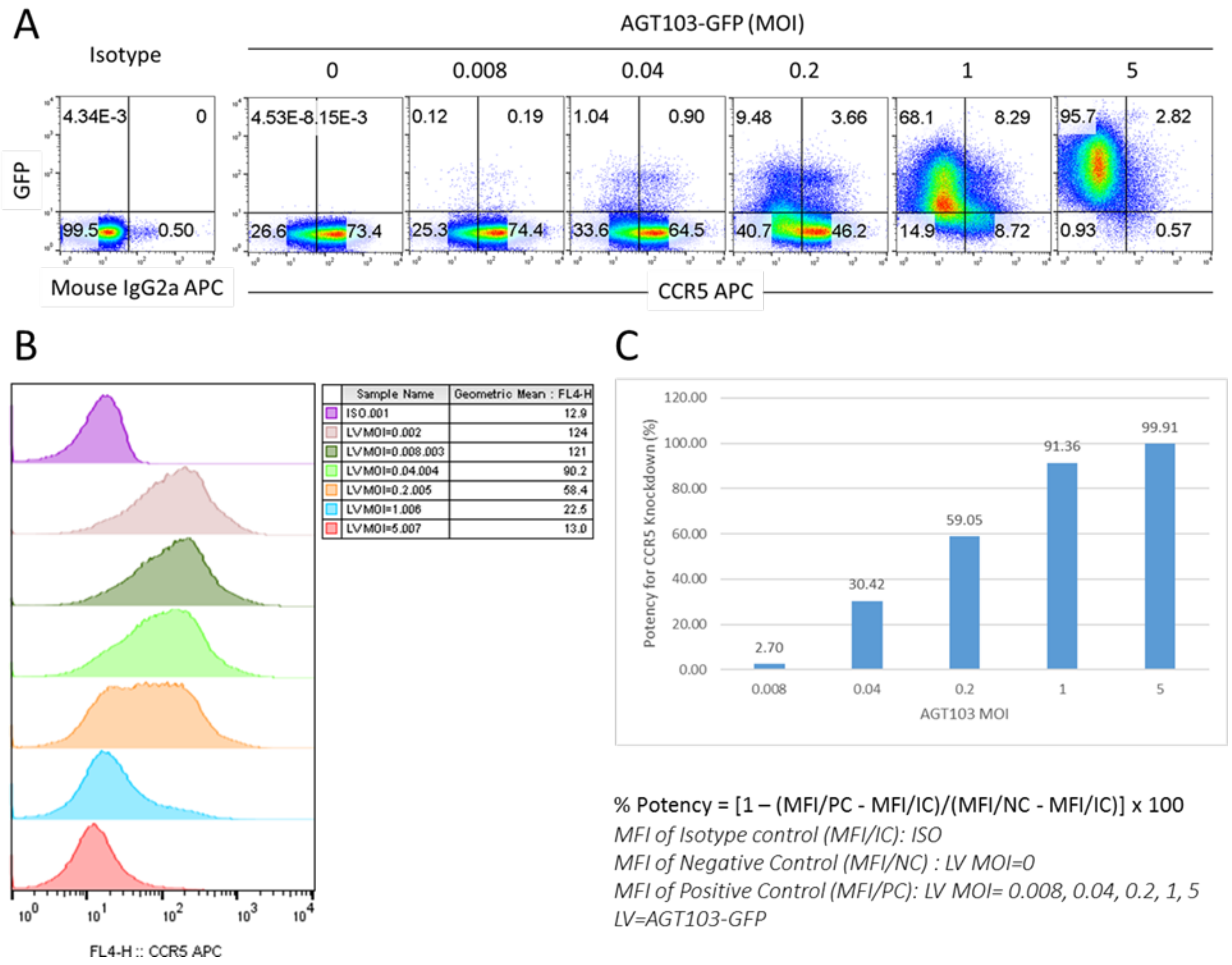

Figure S1. Knockdown of CCR5 by AGT103. CEM.NKR.CCR5 cells were transduced with AGT103-GFP at various doses. Transduction efficiency and CCR5 expression were examined by flow cytometry (A). Mean fluorescence intensity (MFI) was analyzed (B). Potency of CCR5 knockdown was calculated based on the MFI of CCR5 expression (C).

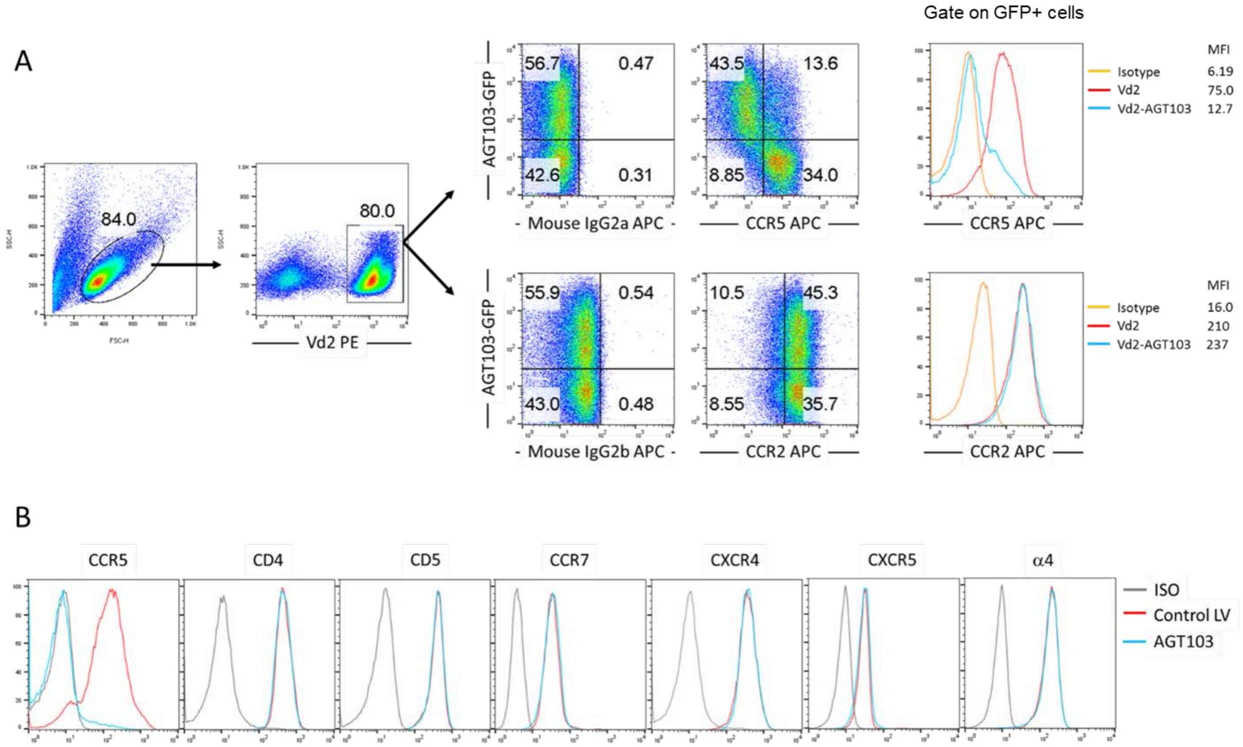

Figure S2. Specificity of AGT103 on CCR5 knockdown. (A) Vd2 T cells were transduced with AGT103-GFP. The expression of CCR5 and CCR2 were examined by flow cytometry. (B)CEM.NKR.CCR5 cells were transduced with AGT103. The expression of CCR5, CD4, CD5, CCR7, CXCR4, CXCR5 and $\alpha4$ were evaluated by flow cytometry.

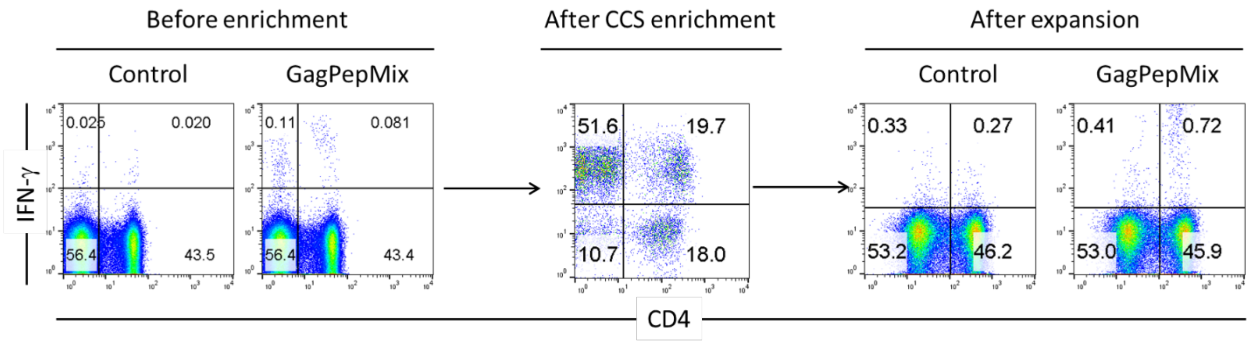

Figure S3. Expansion of HIV Gag-specific CD4 T cells by CCS enrichment and non-specific stimulation. PBMC were stimulated with HIV Gag peptide for 4 hours and IFNγ-positive cells were separated with Miltenyi CCS. The separated cells were rested in medium for 24 hours before TransAct was added. Fresh medium with IL7/IL15 and Saquinavir were added every 2-3 days during the cell expansion. After expansion, Peptide restimulation and an intracellular staining assay was performed to detect IFN-γ positive antigen-specific cells.

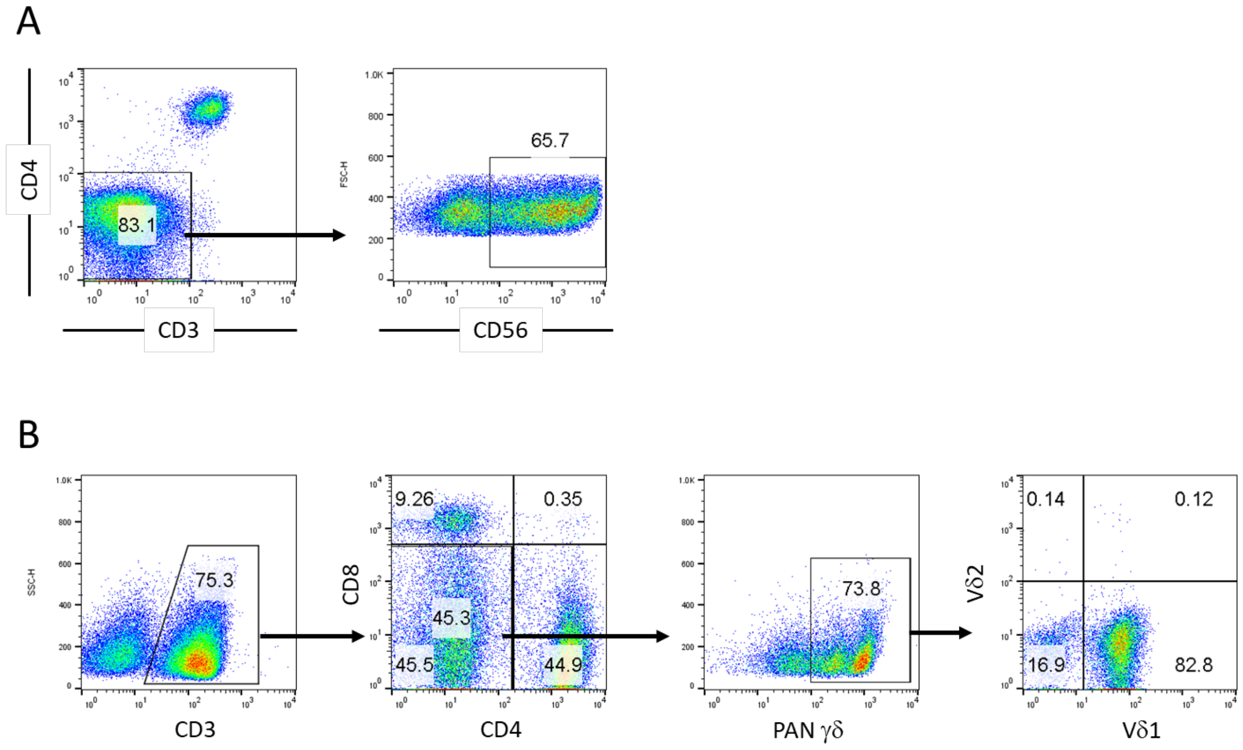

Figure S4. Overgrowth of NK and Vδ1 T cells. (A and B) PBMC were stimulated with HIV Gag peptide for 18 hours. CD8 T cells were depleted. The negative selected cells were expanded in medium containing IL7/IL15 and Saquinavir. After expansion, HIV Gag peptide restimulation and an intracellular staining assay was performed to detect cell subsets and IFN-γ positive antigen-specific cells. NK (A) or Vδ1 (B) cells were expanded in different donors.

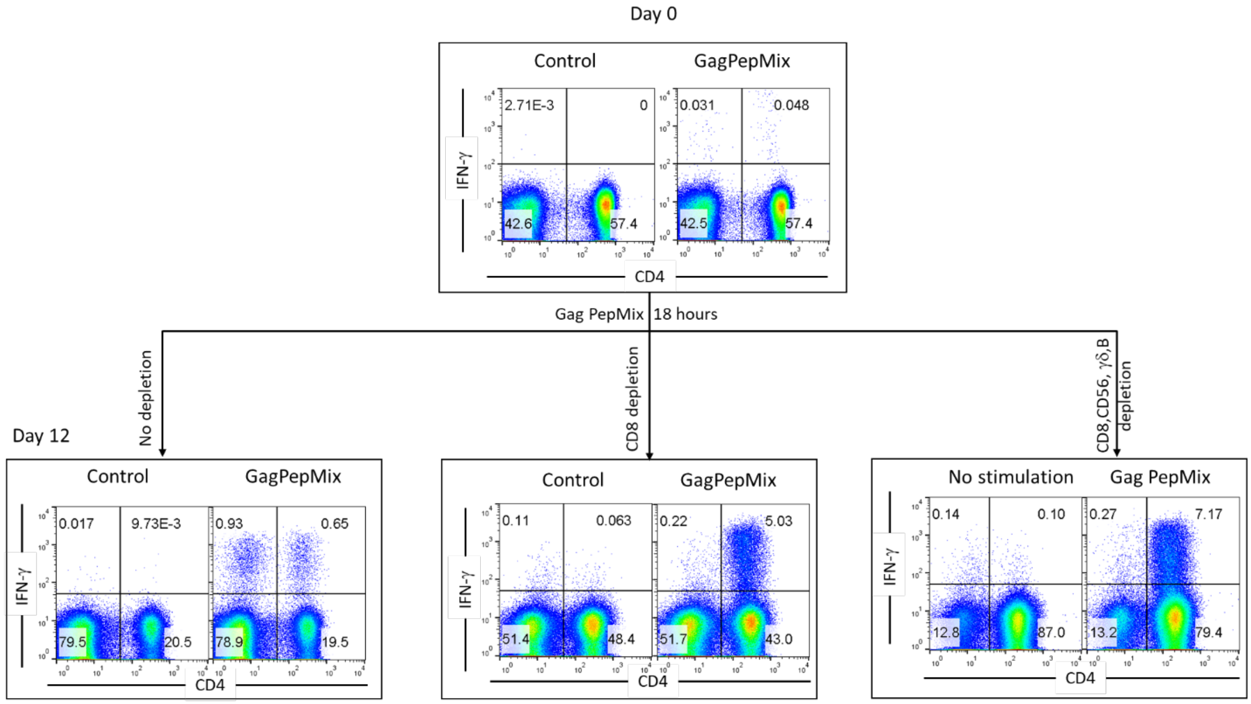

Figure S5. Optimization of HIV Gag-specification CD4 T cell expansion by depletion of other subsets. PBMC were stimulated with HIV Gag peptide for 18 hours. CD8, CD56, γδ or CD19 cells were depleted. The negative selected cells were expanded in medium containing IL7/IL15 and Saquinavir. After expansion, HIV Gag peptide restimulation and an intracellular staining assay was performed to detect cell subsets and IFN-γ positive antigen-specific cells.

Before expansion

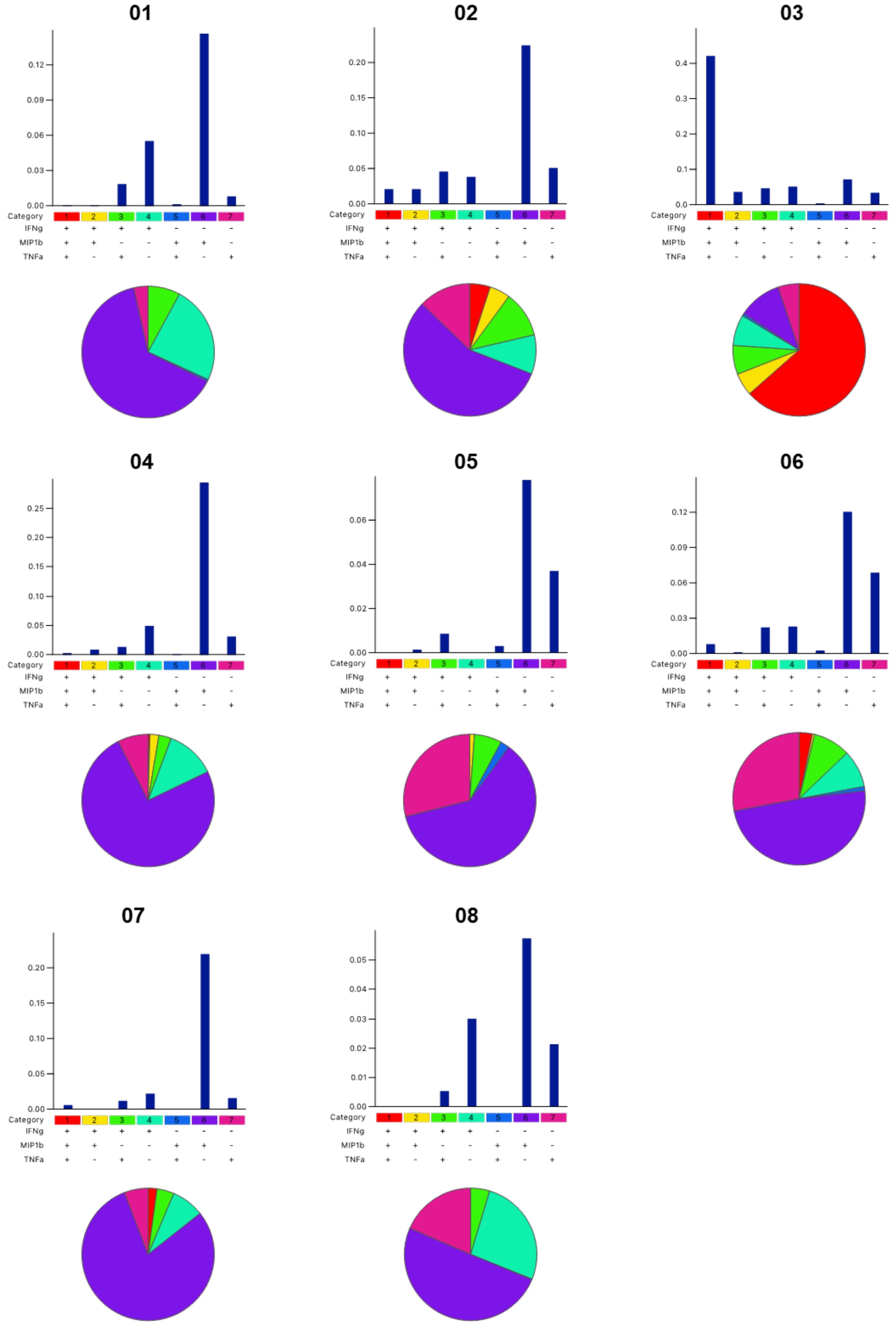

After expansion

01

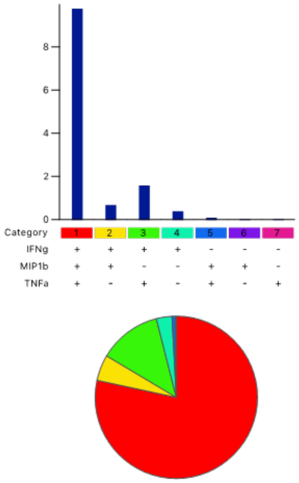

02

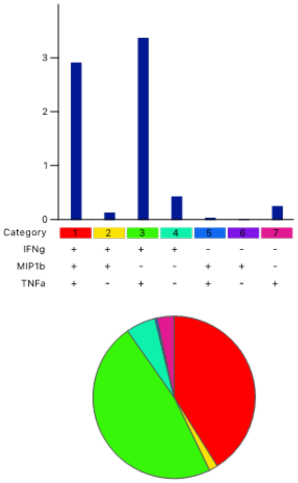

03

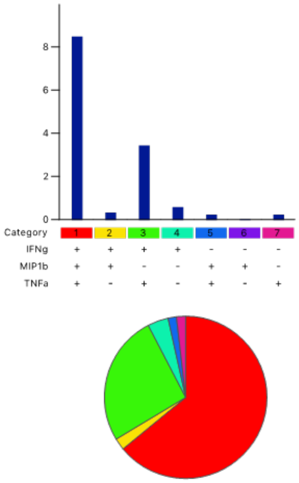

04

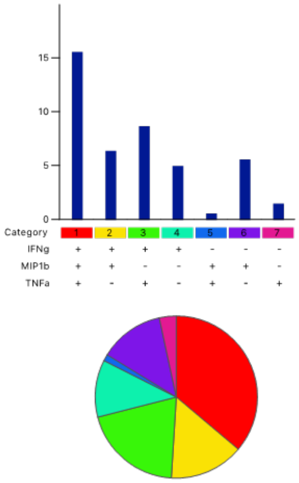

05

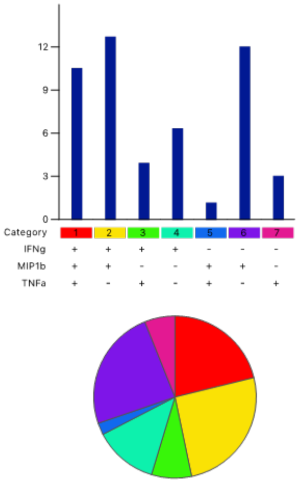

06

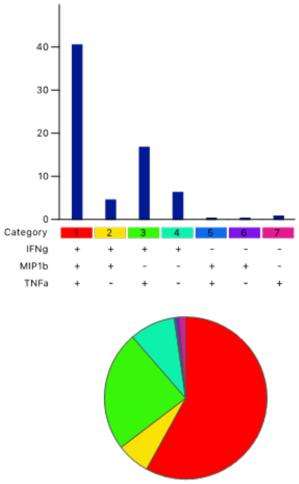

07

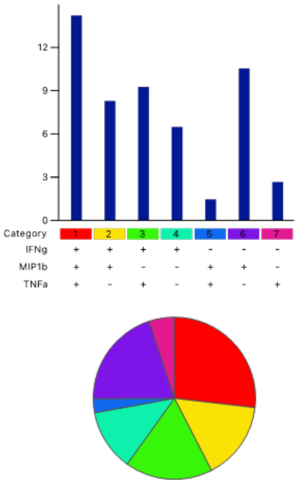

08

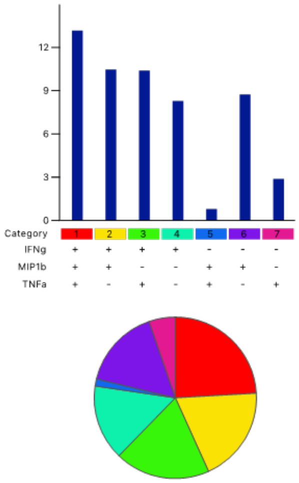

Figure S6. IFN-γ, TNF-α and MIP-1ß analysis simultaneously by multiparameter flow cytometry. Cells before and after expansion were stimulated with Gag peptides. Frequency of CD4 T cells producing different combinations of IFN-γ, TNF-α and MIP-1ß was analyzed. The relative proportions of cells making different cytokines were depicted by pie charts.

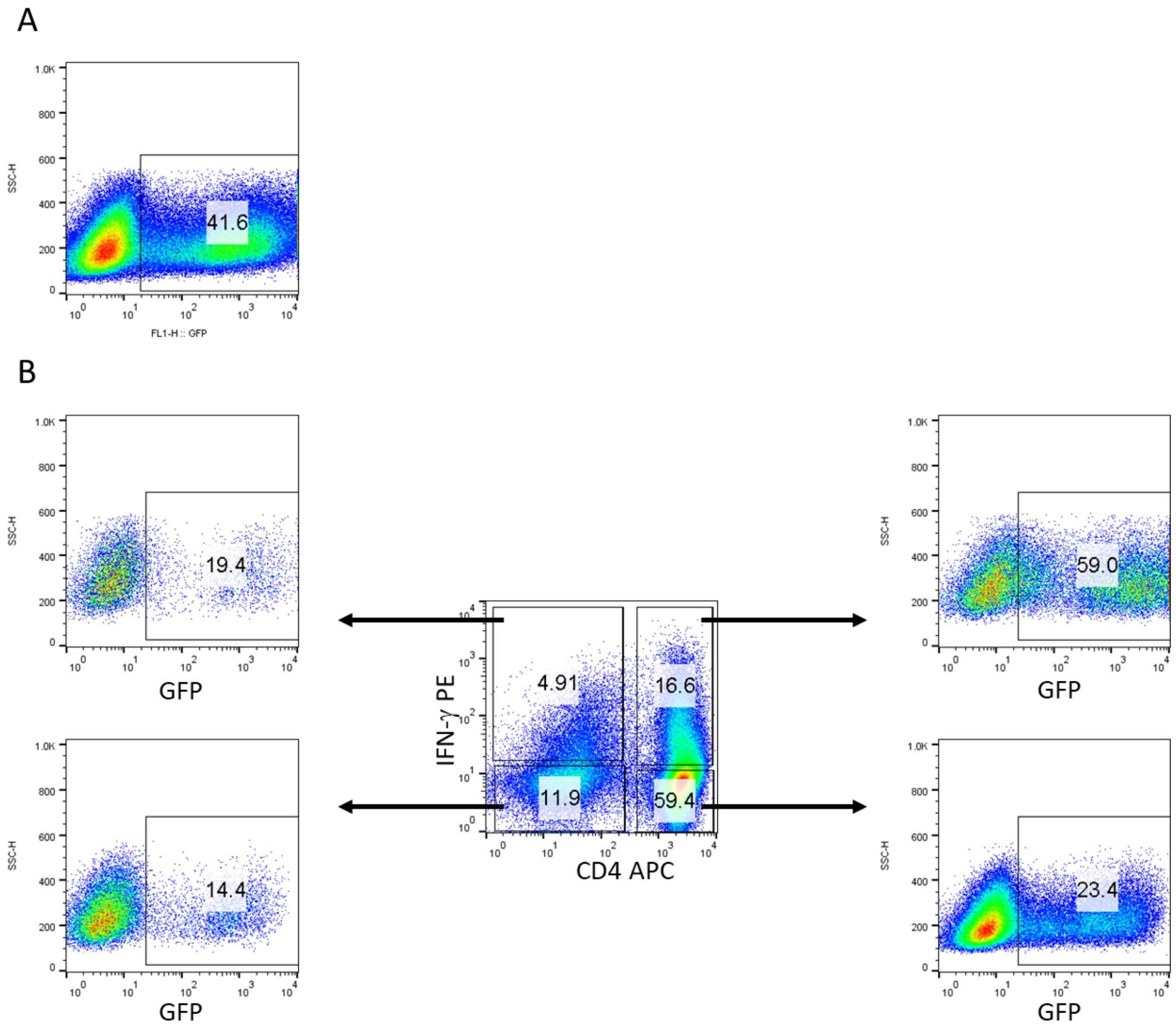

Figure S7. Evaluation of lentivirus transduction efficiency. PBMC were stimulated with HIV Gag peptide for 18 hours. CD8/γδ/NK/B T cells were depleted. The negative selected cells were transduced with AGT103-GFP and expanded in medium containing IL7/IL15 and Saquinavir. After expansion, HIV Gag peptide restimulation and an intracellular staining assay was performed to detect cell IFN-γ positive antigen-specific cells and GFP-positive transduced cells.

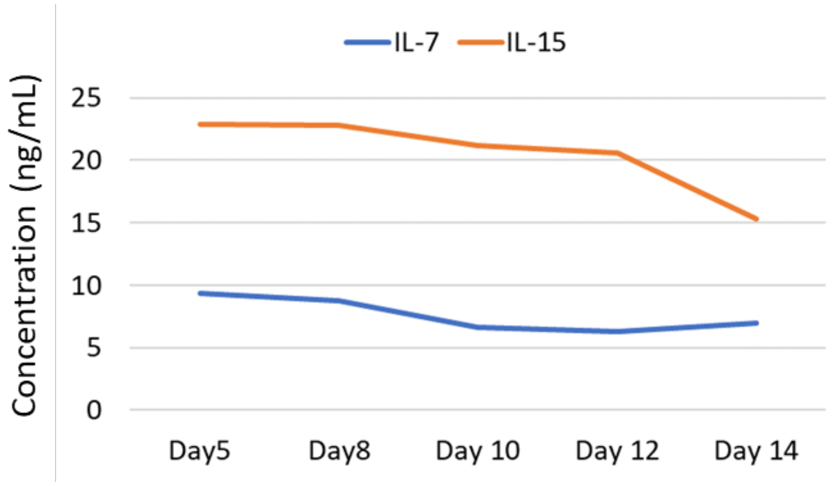

Figure S8. Cytokine stability during cell culture in G-Rex. Determination of cytokine concentration in the culture medium by using hIL-7 and hIL-15 ELISA.

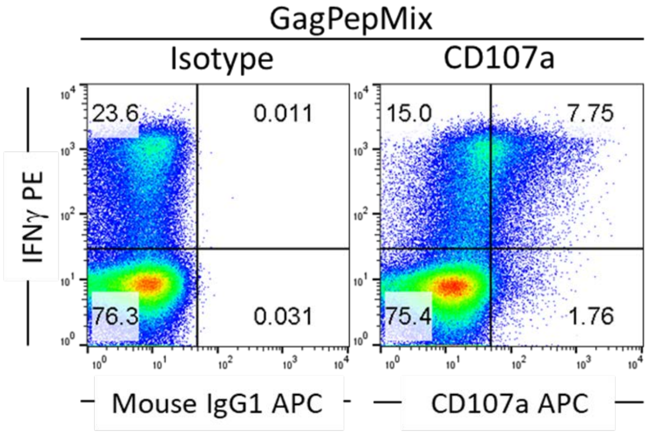

Figure S9. Detection of HIV Gag-specific cytotoxic CD4 T cells. HIV Gag peptide restimulation and an intracellular staining assay was performed to detect CD107a and IFN-γ positive antigen-specific cells.
